# Novel glitazones as PPARγ agonists: molecular design, synthesis, glucose uptake activity and 3D QSAR studies

**DOI:** 10.1186/s13065-018-0508-0

**Published:** 2018-12-19

**Authors:** Subhankar P. Mandal, Aakriti Garg, P. Prabitha, Ashish D. Wadhwani, Laxmi Adhikary, B. R. Prashantha Kumar

**Affiliations:** 1 Department of Pharmaceutical Chemistry, JSS College of Pharmacy, JSS Academy of Higher Education and Research, Mysuru, 570 015 India; 2 Department of Pharmaceutical Biotechnology, JSS College of Pharmacy, Ootacamund, 643 001 India; 3 Bioanalytical Division, Biocon Ltd, Bengaluru, 560 100 India

**Keywords:** PPARγ, Pharmacophore, Molecular docking, Glucose uptake assay, 3D-QSAR, CoMSIA

## Abstract

**Background:**

An alarming requirement for finding newer antidiabetic glitazones as agonists to PPARγ are on its utmost need from past few years as the side effects associated with the available drug therapy is dreadful. In this context, herein, we have made an attempt to develop some novel glitazones as PPARγ agonists, by rational and computer aided drug design approach by implementing the principles of bioisosterism. The designed glitazones are scored for similarity with the developed 3D pharmacophore model and subjected for docking studies against PPARγ proteins. Synthesized by adopting appropriate synthetic methodology and evaluated for in vitro cytotoxicity and glucose uptake assay. Illustrations about the molecular design of glitazones, synthesis, analysis, glucose uptake activity and SAR via 3D QSAR studies are reported.

**Results:**

The computationally designed and synthesized ligands such as *2*-*(4*-*((substituted phenylimino)methyl)phenoxy)acetic acid* derivatives were analysed by IR, ^1^H-NMR, ^13^C-NMR and MS-spectral techniques. The synthesized compounds were evaluated for their in vitro cytotoxicity and glucose uptake assay on 3T3-L1 and L6 cells. Further the activity data was used to develop 3D QSAR model to establish structure activity relationships for glucose uptake activity via CoMSIA studies.

**Conclusion:**

The results of pharmacophore, molecular docking study and in vitro evaluation of synthesized compounds were found to be in good correlation. Specifically,** CPD03, 07, 08, 18, 19, 21** and** 24** are the candidate glitazones exhibited significant glucose uptake activity. 3D-QSAR model revealed the scope for possible further modifications as part of optimisation to find potent anti-diabetic agents. 
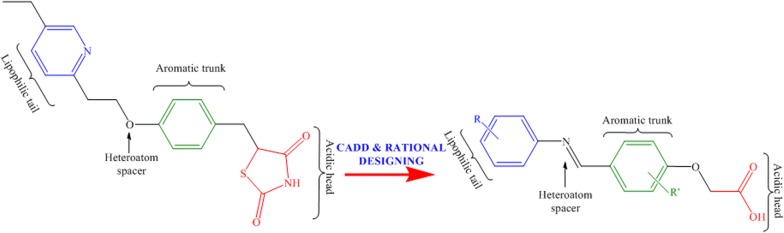

## Introduction

Type 2 Diabetes mellitusis a heterogeneous group of disorders linked to the inability to regulate glucose metabolism. Unfortunately, incidence in individuals is increasing with respect to time [1]. The prevalence of diabetes increases with age and currently affects one-fifth of the world’s population [2, 3]. The mechanism by which the complications of the affected patient’s increases is still unknown, but the most commonly accepted hypothesis is that, Type 2 Diabetes is multifactorial which includes both genetic and environmental elements that affects tissue insulin sensitivity and beta-cell functions. Although it is generally agreed that both has significant roles in plasma glucose regulation, however, the interlinking mechanisms controlling these two impairment is still unknown [4, 5].

Transcription factors such as Peroxisome Proliferator Activated Receptors (PPARs) has been extensively studied and reported for its metabolic functions, which belongs to the nuclear receptor superfamily, whose members possess selectivity towards lipophilic ligands and transduce chemical signals into specific changes in gene expression [6, 7]. There are three PPAR subtypes, as PPARα, PPARβ/δ, and PPARγ; they are closely connected factors which control the midway metabolism of glucose and lipid homeostasis, adipogenesis, immune response, cell growth and differentiation [8, 9]. Out of all, agonistic activation of PPARγ can efficiently regulate plasma glucose level; hence can control Type 2 Diabetes [10].

**Fig. 1:**
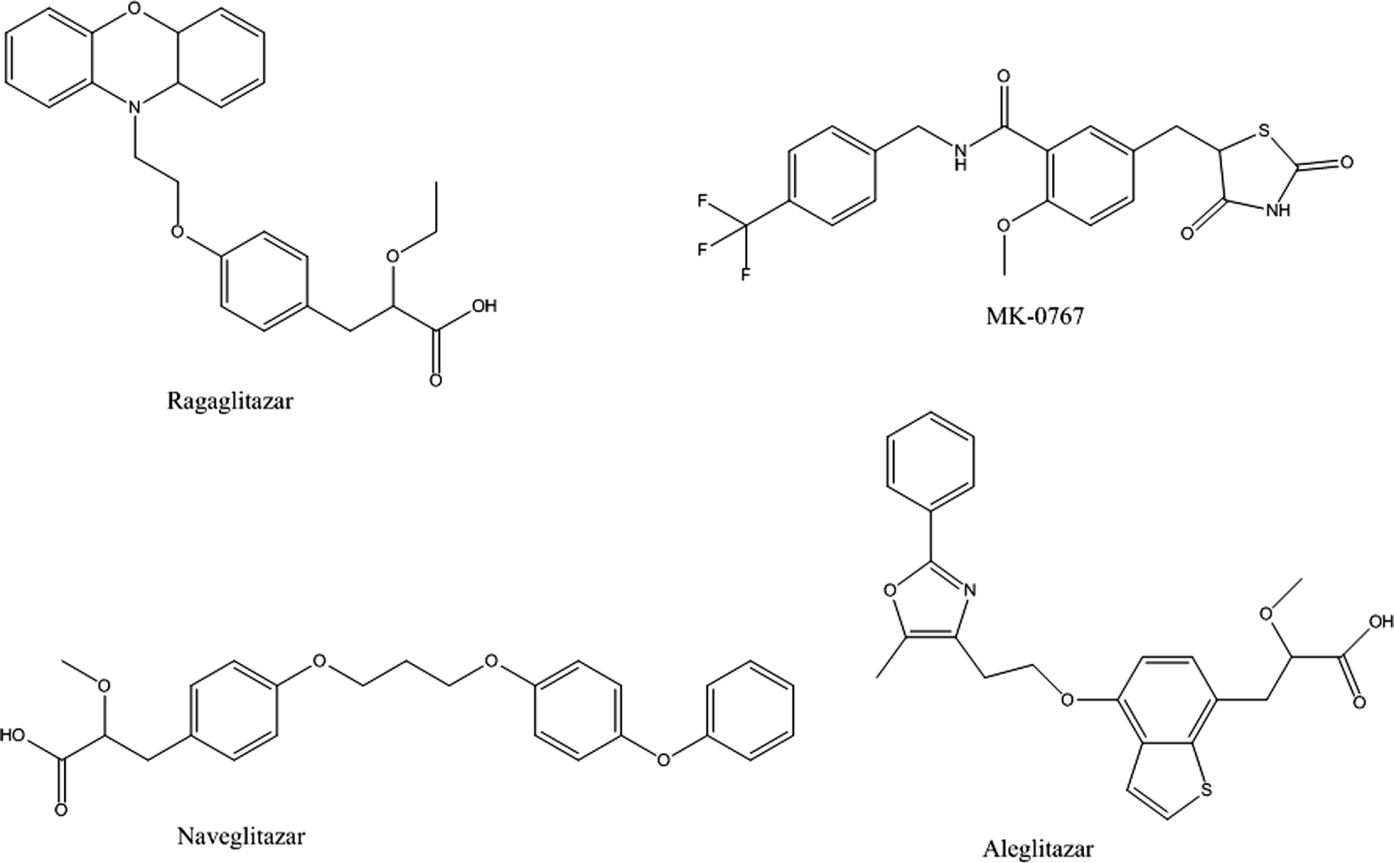
Glitazars with potential antihyperglycemic activity

It is very astounding to notice that in last few decades development of antidiabetic drugs was very profound, and drugs approved by USFDA is gigantic in number, hence there is a persistent need in innovative development of novel antidiabetic drugs [11, 12]. In past, several attempts were made to identify agents with thiazolidinedione, oxazolidinedione, tetrazole, oxathiadiazol and α-alkoxy carboxylic acid derivatives but none of them showed optimum desired activity. Several PPARγ agonists (Glitazones) have been developed (Fig. 1) [13], most explicitly thiazolidinediones, a well-known member of glitazone family were studied and widely used for the above purposes, however, they show some side effects besides being pharmacologically active [14]. Glitazones such as ragaglitazar, MK-0767, aleglitazar and naveglitazar, just to name a few, have exhibited clinical utility to glycaemic control and insulin sensitivity but also found to be associated with an increased incidence of both bladder cancer and hyperplasia in rodent studies [15].

**Fig. 2:**
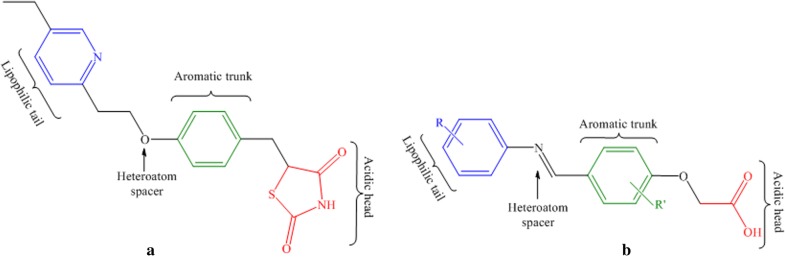
**a** Structural features of pioglitazone; **b** Common structural features of designed glitazone library

Considering the various side effects associated with thiazolidinedione ring, bioisosteric replacement of the ring alongside keeping other structural features intact, such as, aromatic trunk, hetero atom spacer and hydrophobic tail may possibly good (Fig. 2) for antidiabetic, lipid lowering and anti-cancer activities [15–21].

In light of above facts, in the present study, we performed 3D pharmacophore search, molecular docking and dynamics, synthesis and anti-hyperglycemic (glucose uptake) studies on novel glitazones of phenoxy acetic acid derivatives.

## Results and discussion

### Pharmacophore design studies

**Fig. 3:**
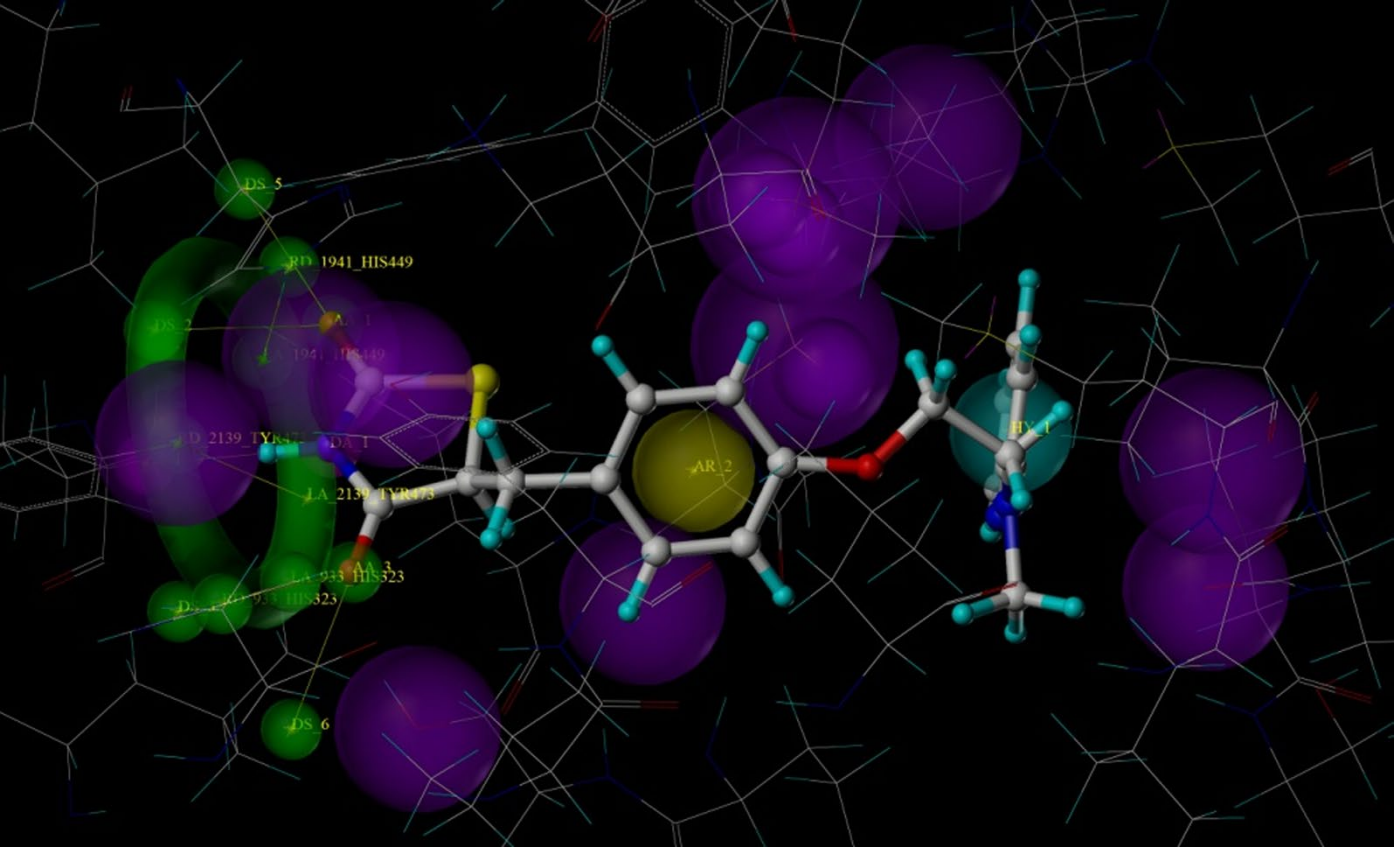
Pharmacophoric QUERY built from protein ligand complex, where green representing hydrogen bond acceptor (0.5 Å tolerance), magenta representing hydrogen bond donor (0.5 Åtolerance) and also neighbouring amino acids (1.0 Å tolerance), yellow representing aromatic ring (1.0 Å tolerance) and cyan representing hydrophobic ring (1.0 Å tolerance)

Pharmacophore based drug design approaches are one of the important tools in drug discovery. Various ligand-based and structure-based virtual screening protocols adopt pharmacophore modelling [22]. Considering importance of pharmacophore in rational drug design, we made an attempt to develop structure-based pharmacophore from PPAR-glitazone bound protein complex by generating pharmacophoric QUERY (Fig. 3). The built QUERY was searched against a dataset of rationally designed glitazones (2234 glitazones), designed from different possible substituent combinations, keeping common structural scaffold intact. For validation of built pharmacophore model, the Güner-Henry (GH) [23] scoring method was adopted, where a decoy set of 1724 molecules were also screened against the built QUERY. Finally, the statistical parameters such as, %AD, %AE, %Y, E and GH were calculated. All the manually designed glitazars along with reference pioglitazone were searched against the 3D pharmacophore QUERY. The % similarity (QFIT) of higher ranked compounds amongst the database searched are as shown in Table 1. The validation of search operation was evaluated by analysing the statistical parametersas shown in Table 2 for the number of hits generated.

**Table 1:** QFIT scores of the designed glitazones

Sl. no.	Compound	QFIT	Sl. no.	Compound	QFIT
1.	Pioglitazone	96.24	14	CPD16	61.56
2.	CPD19	86.45	15	CPD24	60.30
3.	CPD07	86.45	16	CPD22	60.00
4.	CPD21	86.45	17	CPD10	58.29
5.	CPD03	85.31	18	CPD13	58.02
6.	CPD20	85.12	19	CPD23	55.03
7.	CPD08	85.06	20	CPD17	54.67
8.	CPD14	81.29	21	CPD09	54.67
9.	CPD12	74.24	22	CPD15	52.33
10.	CPD11	74.24	23	CPD06	52.33
11.	CPD04	74.24	24	CPD02	50.45
12.	CPD05	68.29	25	CPD01	50.45
13.	CPD18	61.56	–	–	–

To validate the derived pharmacophore model decoy set of 1724 molecules from ZINC databasewere searched against built pharmacophore QUERY. Finally the number of hits was calculated by considering the QFIT > 50

Number of hits (H_a_) obtained from active dataset = 24

Number of hits (H_d_) obtained from decoy dataset = 12

Number of actives (A) = 2234, number of decoy (D) = 1724

**Table 2:** Statistical parameters of pharmacophore study

Sl no.	Statistical parameters	Value obtained
1.	% AD (percentage of actives in the dataset)	1.43%
2.	% Y (percentage of actives in total hits)	45.45%
3.	% AH (percentage of actives returned as hits)	40%
4.	E (enrichment factor by which results are richer in actives than dataset)	31.78
5.	GH (Güner-Henry) score	6.12

The 3D pharmacophore based search operation identified the possible hits among the whole dataset by considering QUERY structural features. The QFIT value of pioglitazone was found to be highest among all because the QUERY was generated from the proteinbound to pioglitazone as reference ligand; this eventually reflects the quality of pharmacophore model. The database search identified** CPD03, 07, 08, 19** and** 21** as possible hits among the whole dataset of glitazones. The statistical parameters such as  % AD,  % Y,  % AH, E and GH represents the quality of pharmacophore model, especially the GH score between 6 to 10 indicates good pharmacophore model thereby its enhanced predictability.

### Docking study

**Fig. 4:**
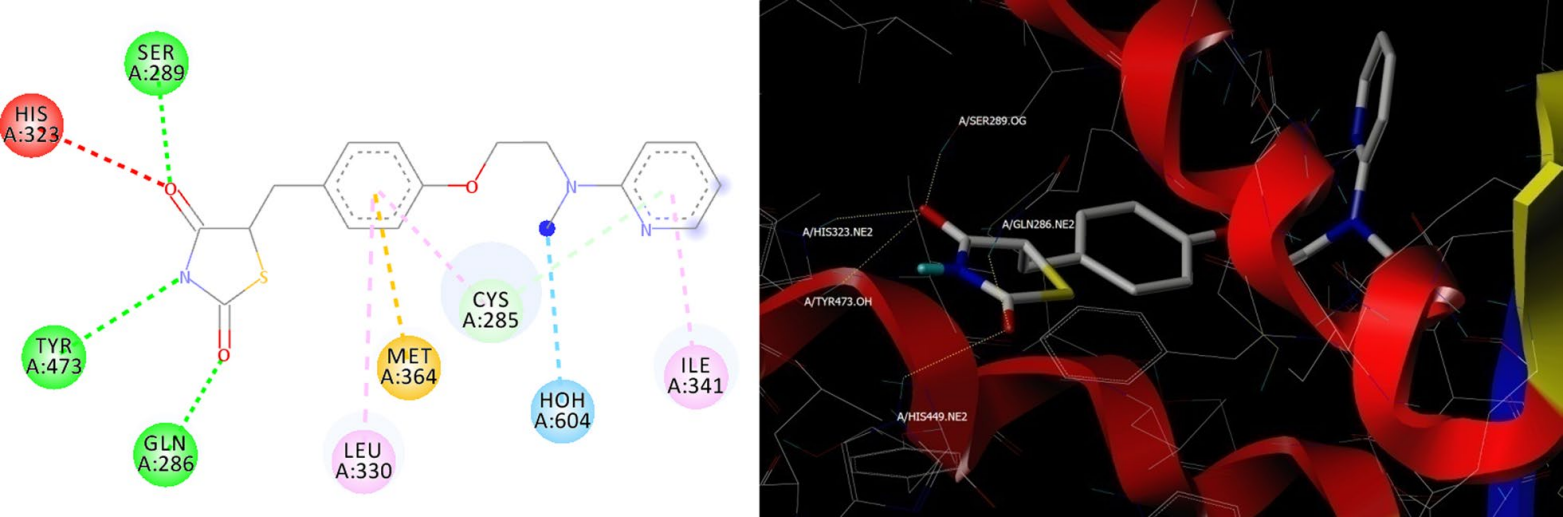
Binding pose of reference ligand (pioglitazone) present in PPARγ (PDB code: 3CS8) before (2D) and after (3D) Docking studies, His 323, His 449, Tyr 473, Ser 289 and Gln 286 are important binding pocket amino acids to form hydrogen bonds (yellow dots)

**Fig. 5:**
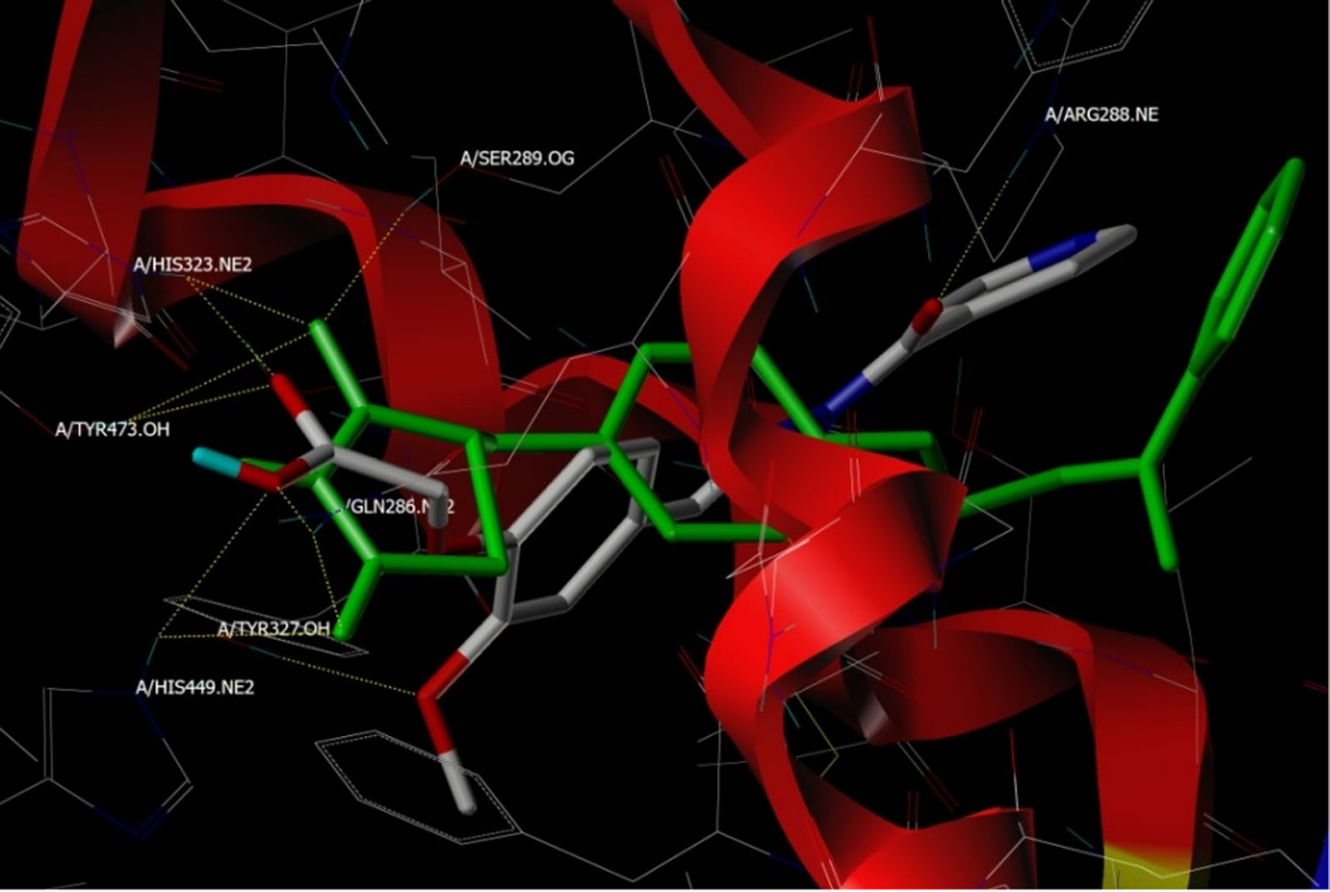
Overlap of pioglitazone (green color) and CPD20 (atom type color) at the binding site of PPARγ showing comparable hydrogen bonding (yellow dots) interactions with amino acids; His 323, Tyr 473, Tyr 327, Gln 286, His 449 and Arg 288 at the binding site

Molecular docking studies virtually defines the binding modes of ligand interaction at the active site of the receptor [24]. Therefore, top 24 hits from the pharmacophore search operation were subjected for docking studies against PPARγ, as part of structure based virtual screening. We performed molecular docking study on the target protein and result is as depicted in Table 3. To validate the docking protocol co-crystalized ligand was checked by re-docking before and after the docking operation [25]. Pioglitazone along with other designed compounds made to bind to the active site of the PPARγ protein. As part of binding interactions with glitazones, important amino acids, such as, His 323, His 449, Tyr 473, Ser 289 and Gln 286 are interacting residues for PPARγ respectively (Fig. 4 and Fig. 5), with Hydrogen bond distances ranged between 2.089 to 3.278 Å.

**Table 3:** Docking scores of compounds with respect to PPARγ protein

Sl. no.	Compound	Total score	Crash score	Polar score
1.	CPD19	7.7081	− 1.3004	2.3039
2.	CPD20	7.5315	− 0.5913	5.4173
3.	CPD22	7.3343	− 1.6674	4.0673
4.	CPD07	7.2051	− 1.9381	4.7489
5.	CPD14	7.8855	− 1.6489	3.761
6.	Pioglitazone	7.7679	− 0.692	5.0003
7.	CPD24	7.7392	− 0.5356	3.7936
8.	CPD15	7.6704	− 0.4739	4.1586
9.	CPD13	7.6558	− 0.6428	3.1524
10.	CPD21	7.1337	− 0.7975	3.6808
11.	CPD03	6.5258	− 0.7834	3.645
12.	CPD16	5.4123	− 0.6506	3.0827
13.	CPD18	5.4092	− 0.4971	3.0934
14.	CPD09	5.3794	− 2.0629	5.5779
15.	CPD04	5.0546	− 0.8747	3.314
16.	CPD17	4.9768	− 0.5406	2.5312
17.	CPD06	4.8464	− 0.4294	2.7807
18.	CPD12	4.7746	− 0.2911	3.6919
19.	CPD02	4.3294	− 0.5312	3.2295
20.	CPD23	4.2525	− 0.3915	2.3399
21.	CPD05	4.2156	− 1.7538	2.8872
22.	CPD10	3.9722	− 0.6071	1.9545
23.	CPD11	3.8578	− 1.0167	3.0595
24.	CPD01	3.8079	− 0.7685	3.1815
25.	CPD08	3.6961	− 0.5147	3.4152

Crash score: revealing the inappropriate penetration into the binding site

Polar score: reports the polar region of the ligands

### Molecular dynamic simulation studies

**Fig. 6:**
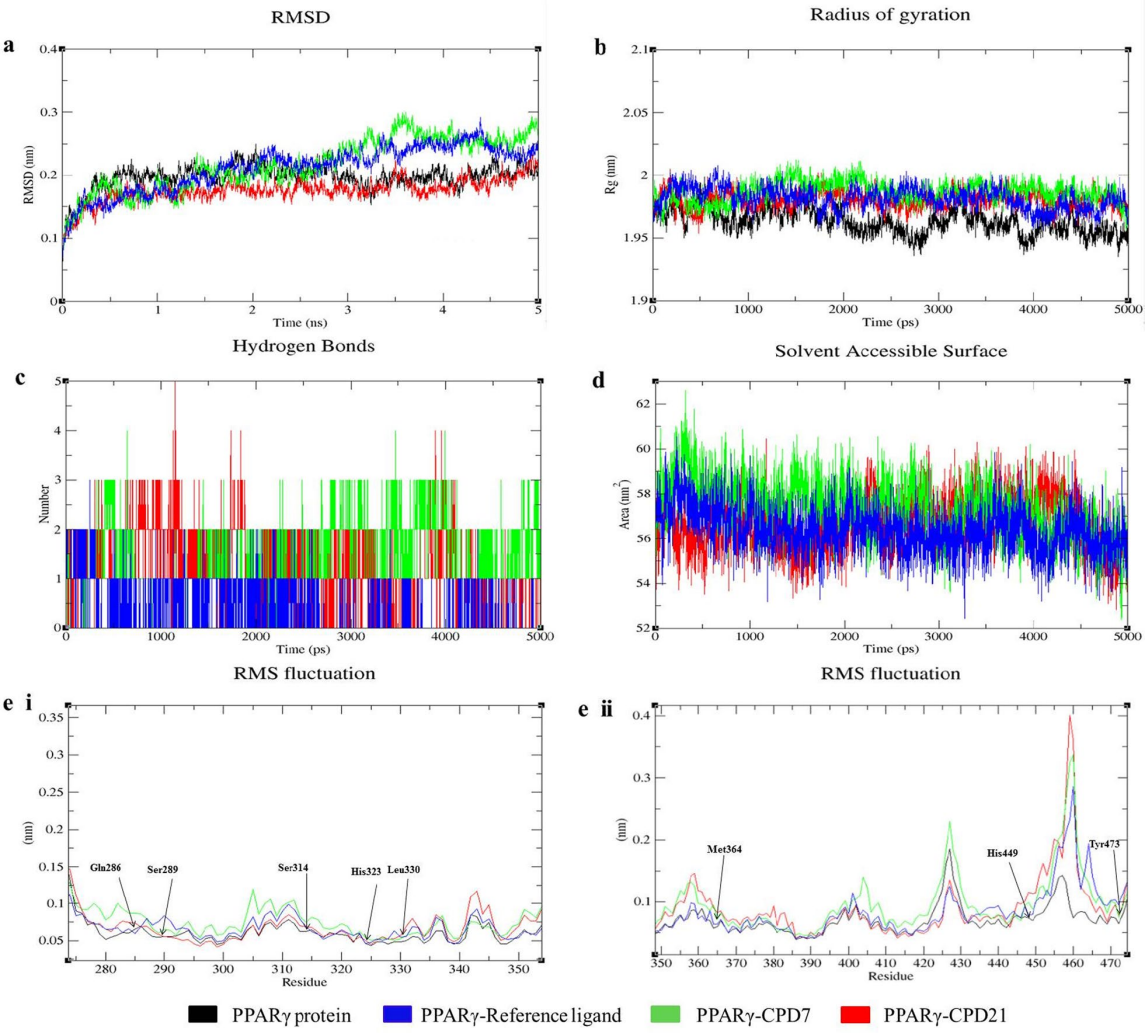
Analysis of RMSD, *R*_*g*_, hydrogen bond, SASA and RMSF of PPARγ with reference ligand, CPD 07 and CPD 21 complexes at 5000 ps. **a** Time evolution of backbone RMSD of the PPARγ protein alone and with reference ligand, CPD 07 and CPD 21 complex structures. **b** Radius of gyration (*R*_*g*_) of the protein backbone in its free and complex form over the entire simulation time. The ordinate is *R*_*g*_ (nm) and abscissa is time (ps) interval. **c** Hydrogen bonds occurring over the time of simulation between protein and different ligands. **d** SASA is indicated, where ordinate is SASA (nm) and abscissa is time (ps). **e**. I and II Residue wise average RMSF plot of protein and different ligands

Molecular dynamic (MD) simulation studies is ideal to perform after the docking studies to understand the dynamic behaviour of the protein–ligand complex conformationsin order to mimic the behaviour in actual environment [26]. It provides detailed information on the motion of whole molecule as well as individual atoms as a function of time and thereby provides valuable information between them [27]. In this context, we performed MD simulation to understand the binding affinities of different ligands (reference ligand,** CPD07**, and** CPD21**) with PPARγ protein in its free and in the form of complex. To make a comparative understanding of the dynamic behavior of our designed ligands with the target proteins, we took reference ligand whose stability with the proteins in the complex is well known. We have analysed root mean square deviation (RMSD), root mean square fluctuation (RMSF), radius of gyration (Rg), solvent accessible surface area (SASA), number of hydrogen bonds, and variation of secondary structure pattern between the protein and their complexes (PPARγ with reference ligand,** CPD07** and** CPD21**). Four independent simulations were carried out for the native protein structure along with their respective complexes for 5 ns simulation time. We found that the protein, PPARγ, in its free and complex form reach equilibrium approximately at 2 ns of time and then after showing stable trajectory and resonates only between 0.10 to 0.25 nm of RMSD till the end of the remaining simulation (Fig. 6a), it’s been clear from the RMSD plot that PPARγ is quite flexible in its free form and deviating in reasonably higher RMSD values than its complex forms (Fig. 6a), led to the conclusion that the complex formation influenced changes in the flexibility of the dynamic behavior of the protein. We have also observed that** CPD21** in its complex with protein have very narrow RMSD value (0.15–0.20 nm), hence predicted to form stable complexes till the end of the simulation. Similarly, we have observed that** CPD07** with the protein showed similar trajectories but minimal deviations were observed from the perspective of equilibration time and average RMSD values, although attaining final stable equilibration through to the end of the simulation. All the complexes throughout the simulation tend to reach a stable trajectory. The higher RMSD obtained for all complexes limited to 0.3 nm exhibits that the simulations produced stable trajectories and delivered an appropriate root for further investigation.

The radius of gyration calculates the mass weighted root mean square distance of atoms from their respective centre of mass. The overall structures at various time points during the trajectory can be analysed for capability, shape and folding in the plot of *R*_*g*_ (Fig. 6b). Throughout the simulation, all the proteins and their complexes exhibited a similar pattern for the *R*_*g*_ value, at which *R*_*g*_ score for PPARγ group ranges at higher value of 1.95–2.00 nm.

The number of hydrogen bonds formed between different residues of protein and ligands during the course of the simulation was also calculated (Fig. 6c). From the observed graph, it is understood that the different complexes formed a different number of hydrogen bonds, with an average value of ~ 0–3 number. For comparison, reference ligand formed extra number of hydrogen bond than **CPD07 **and** CPD21**. It is interesting to observe that the number of hydrogen bond (NH) formed and maintained during the MD simulation is in very much consistent with the docking results, but the fluctuation in the number of hydrogen bond can only be explained by the dynamic movement of the protein and interacting ligands during the simulation time, which may introduce or omitted few hydrogen bonds over the whole simulation time.

To measure the compactness of the hydrophobic core forming between different protein–ligand complexes, SASA (solvent accessible surface area) were measured (Fig. 6d). Results show diversity in SASA values, observed with different PPARγ complexes, particularly PPARγ-**CPD07** complex shows higher values of SASA (56–60 nm), whereas** CPD21** and reference ligand with PPARγ complex show lower SASA value (55–59 nm).

Further, C-RMSF is calculated to observe the overall flexibility of atomic positions in the trajectory for the proteins and their complexes (Fig. 6e. I and e. II). The PPARγ protein with** CPD07** and** CPD21** showed a significant change in protein structure conformation with an increase in the C-RMSF values but interestingly the active site amino acid residues such as, Gln286, Ser289, Ser314, His323, Lue330, Met364 and His449 fluctuated at very narrow range, indicating the protein structure conformation is conserved.

### Chemistry and synthesis

**Scheme 1:**
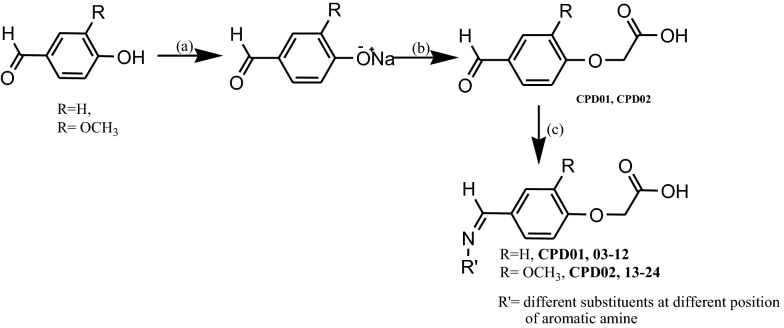
Synthesis of target phenoxyacetic acid based glitazones **CPD01-24**

Rationally designed target compounds belonging to the class of 2-(4-((substituted phenylimino)methyl)phenoxy)acetic acid; (**CPD01-24**) were synthesized according to the Scheme 1 [28, 29]. Two aromatic aldehydes, namely, viz. 4-hydroxy benzaldehyde and 4-hydroxy-3-methoxy benzaldehyde (vanillin) were selected as building blocks. In the first and second step of total synthesis, we have converted hydroxyl group of aldehyde to corresponding phenoxy-acetic acids by adopting the modified procedure of Williamson ether synthesis [30, 31]. Further, the aldehyde functional group was condensed with primary aromatic amines to form Schiff base via imine linkage [32–35]. From our observational perspective it was found that, phenoxy-acetic acid moiety of intermediates hinders the interaction of aldehyde and amines of different reactants and hence formation of imine linkages also deters. The structures of the synthesized glitazars confirmed via IR, NMR and Mass spectral interpretation, data of respective studies are provided at experimental section.

The appearance of characteristic peak in the range of 1580.50 to 1604.90 cm^−1^ in all IR spectra along with the absence of NH stretch proved the formation of imine bond (CH=N). All the compounds showed characteristic C=O stretching of carboxyl group in the range of 1650.42 to 1743.35 cm^−1^ along with O–H stretching in the range of 3200.25 to 3400.23 cm^−1^.

From ^1^H-NMR spectra it is observed that methylene protons (CH_2_), which are bridge between phenoxy and carboxylic acid moiety appeared as singlet in the range of δ 4.45 ppm to δ 4.68 ppm and proton attached to imine linkage (H–C=N) of Schiff base has resonated between δ 8.23 ppm to δ 8.98 ppm which intern confirmed the formation of imine. It is very interesting to notice that** CPD06, 09, 10, 16, 19** and** 20** showed, –CH signal of imine (–CH=N) deshielded to the δ ppm 8.9 and above, which is possible when the configuration at –CH=N linkage is ‘E’ form.

**Fig. 7:**
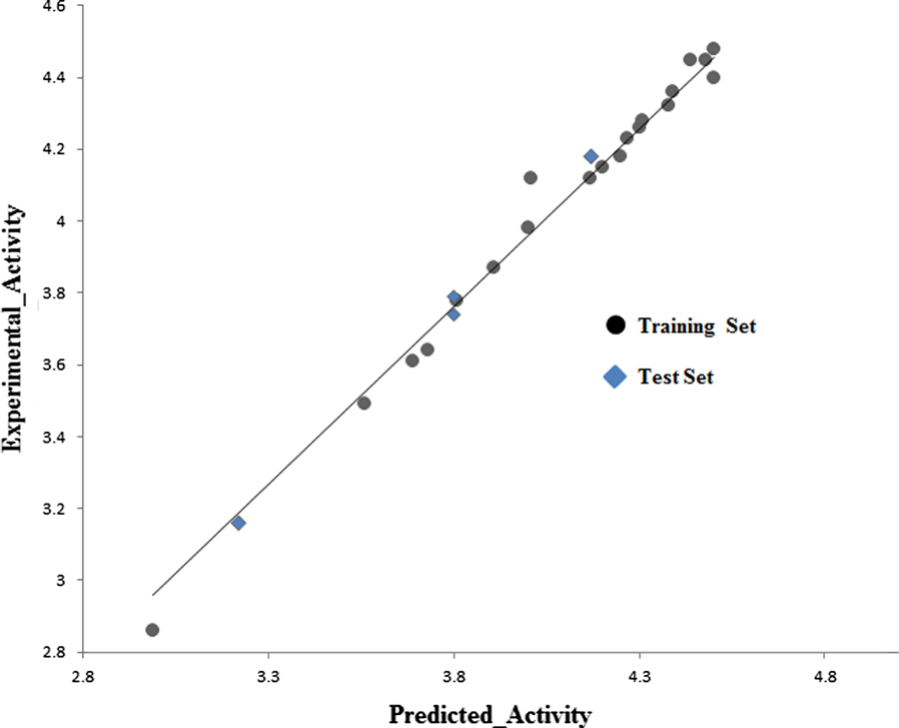
Plot of experimental vs. predicted activity for CoMSIA model of glucose uptake activity

The first step of synthesis involves salt formation of the hydroxyl group by stirring with sodium hydroxide in aqueous medium, further, condensation of the phenoxide sodium with equivalent amounts of chloro acetic acid were done in presence of sodium hydroxide. The method adopted to synthesize the phenoxyacetic acid was very useful as it yields 70–80% of the product with a little modification to the Williamson ether synthesis. Finally, the formed intermediate was utilized to unite to the lipophilic tail by condensing the aldehydic moiety with different substituted aromatic amines using absolute alcohol as solvent in the presence of catalytic amount of acetic acid and few beads of activated molecular sieves. Final products were purified by column chromatography using ethyl acetate and *n*-hexane as solvent system by gradient elution technique.

Reagents and Condition: (a) NaOH, H_2_O, stir (b) ClCH_2_COOH, NaOH, H_2_O reflux at 120–140 °C for 3 h (c) Substituted aromatic amine, gl. acetic acid, absolute ethanol, molecular sieves, reflux with stirring for 8–12 h.

### Biological screening

To evaluate the biological activity of the synthesized compounds, as a first measure, we screened for cytotoxicity followed by glucose uptake assays.

### In vitro cytotoxic assay

All the designed and synthesized 24 compounds were undergone MTT assay to evaluate the cytotoxic concentration levels [36]. Measurement of cytotoxic concentration is always a prerequisite for any in vitro assay. The assay depends both on the number of cells live and dead. The cleavage of MTT to a blue formazan derivative by live cellsby the influence of test compounds, is clearly a very effective means of measuring cytotoxicity [37]. The principle involved is the cleavage of tetrazolium salt MTT (3-(4,5 dimethyl thiazole-2 yl)-2,5-diphenyl tetrazolium bromide) into a blue coloured product (formazan) by mitochondrial enzyme succinate dehydrogenase. The results obtained for cytotoxic assay is as shown in Table 4.

**Table 4:** Cytotoxicity data for the synthesized compounds against 3T3-L1 cells

Sl. no.	Compound	CTC_50_ µg/ml
1.	CPD01	110.78
2.	CPD02	44.16
3.	CPD03	88.38
4.	CPD04	223.59
5.	CPD05	126.28
6.	CPD06	398.59
7.	CPD07	15.87
8.	CPD08	114.86
9.	CPD09	155.65
10.	CPD10	150.86
11.	CPD11	102.62
12.	CPD12	109.88
13.	CPD13	151.86
14.	CPD14	121.98
15.	CPD15	91.98
16.	CPD16	19.95
17.	CPD17	101.63
18.	CPD18	21.15
19.	CPD19	125.19
20.	CPD20	128.36
21.	CPD21	101.37
22.	CPD22	68.92
23.	CPD23	61.71
24.	CPD24	43.41
25.	Pioglitazone	17.56

Cytotoxicity results revealed the different cytotoxic concentration levels of synthesized compounds which are in the range of 15.87 to 398.59 µg/ml with respect to the standard pioglitazone (CTC_50_ 17.56 µg/ml). Cytotoxicity results reveal that the designed molecules are less toxic to the cells when compared with the standard pioglitazone.

### In vitro glucose uptake assay

The skeletal muscles account for more than 80% of insulin-stimulated glucose uptake, an impaired glucose uptake in skeletal muscle is responsible for the development of type II diabetes mellitus [38]. The initial rate-limiting step for glucose clearance in skeletal muscle and adipose tissue is the transport of glucose through a family of specific glucose transporters (GLUT) [38] that are either constitutively presents in plasma membrane or actively translocated to the plasma membrane. A skeletal muscle expresses GLUT1 and GLUT4 glucose transporters. GLUT4 is the main glucose carrier expressed in skeletal muscle, whereas GLUT1 accounts for only 5–10% of total glucose carrier. The regulation of glucose and insulin of the muscle, specific facilitative glucose transport system GLUT4 was investigated in L6 muscle cells in culture [39, 40]. The percentage of glucose uptake activity or anti-diabetic activity for test samples was calculated which is depicted in Table 5.

**Table 5:** Glucose uptake activity of the synthesized compounds

Sl. no.	Compound	% glucose uptake overcontrol
1.	Standard 1	89
2.	Standard 2	94
3.	CPD01	45
4.	CPD02	50
5.	CPD03	81
6.	CPD04	74
7.	CPD05	67
8.	CPD06	45
9.	CPD07	89
10.	CPD08	90
11.	CPD09	20
12.	CPD10	42
13.	CPD11	75
14.	CPD12	72
15.	CPD13	25
16.	CPD14	70
17.	CPD15	45
18.	CPD16	55
19.	CPD17	35
20.	CPD18	65
21.	CPD19	90
22.	CPD20	80
23.	CPD21	85
24.	CPD22	55
25.	CPD23	40
26.	CPD24	70

In vitro glucose uptake activity results indicate that** CPD03, 07, 08, 12, 19, 20** and** 21** has almost identical activity even when compared to standard drug. Other compounds have shown low to moderate glucose uptake activity. Most of the compounds having Vanillin moiety as their trunk showed good activity when compared to others. This could possibly because of additional methoxy group attached to the aromatic ring and supposedly same observations were noted from docking results as well; indicating some apparent correlation between the structures, docking results and the glucose uptake activity.

## 3D QSAR (CoMSIA) studies

Considering the structural diversity of synthesized glitazones and their glucose uptake activity through PPARγ agonistic property; we subjected the group for 3D QSAR studies.

Computational method like Comparative Molecular Similarity Indices Analysis (CoMSIA) [41] was adopted to study the 3D QSAR. This methodology enables us to understand the structural features in 3D space that required to show the activity and also to bind to target receptors. Another advantage of performing CoMSIA study is for contour maps which highlights the structural features and give indication for optimizable areas of the given set of structures to design better active novel molecules [42].

The % glucose uptake values were transferred to their natural logarithms and used for building the model and analysis. The protocol used here are according to the default settings and standard protocol, unless otherwise noted. The cross-validated correlation coefficient (q^2^) value, number of components, non-cross-validated correlation coefficient (r^2^), standard error of estimation (SEE), Fischer’s covariance ratio (F) and contribution of each field components for the developed CoMSIA model areas shown in Table 6. The statistical parameters of the model indicated good predictive ability of developed model.

**Table 6:** Statistical parameters of developed CoMSIA model for glucose uptakeactivity

Statistics	Glucose uptake activity
Grid spacing (Å)	2.0
q^2^ (LOO)	0.712
r^2^	0.889
S	0.211
F	121
No. of components	6
Contribution of-	
Steric	0.080
Electrostatic	0.062
Hydrophobic	0.322
Hydrogen bond donor	0.234
Hydrogen bond acceptor	0.302

q^2^: cross-validated correlation coefficient

r^2^: non-cross-validated correlation coefficient

S: standard error of estimation; F: Fischer’s covariance ratio

The validation and robustness of the developed model was assured by the good q^2^ values (q^2^ > 0.5) [43], hence an external set is used to validate the predictivity of the developed model, whereas the correlation graph (Fig. 7) of predicted activity vs actual activity of training and test set signifies the good regression. 

R = -H for ** CPD01, 03–12**

R = -OCH_3_ for** CPD 02, 13–24**

**Fig. 8:**
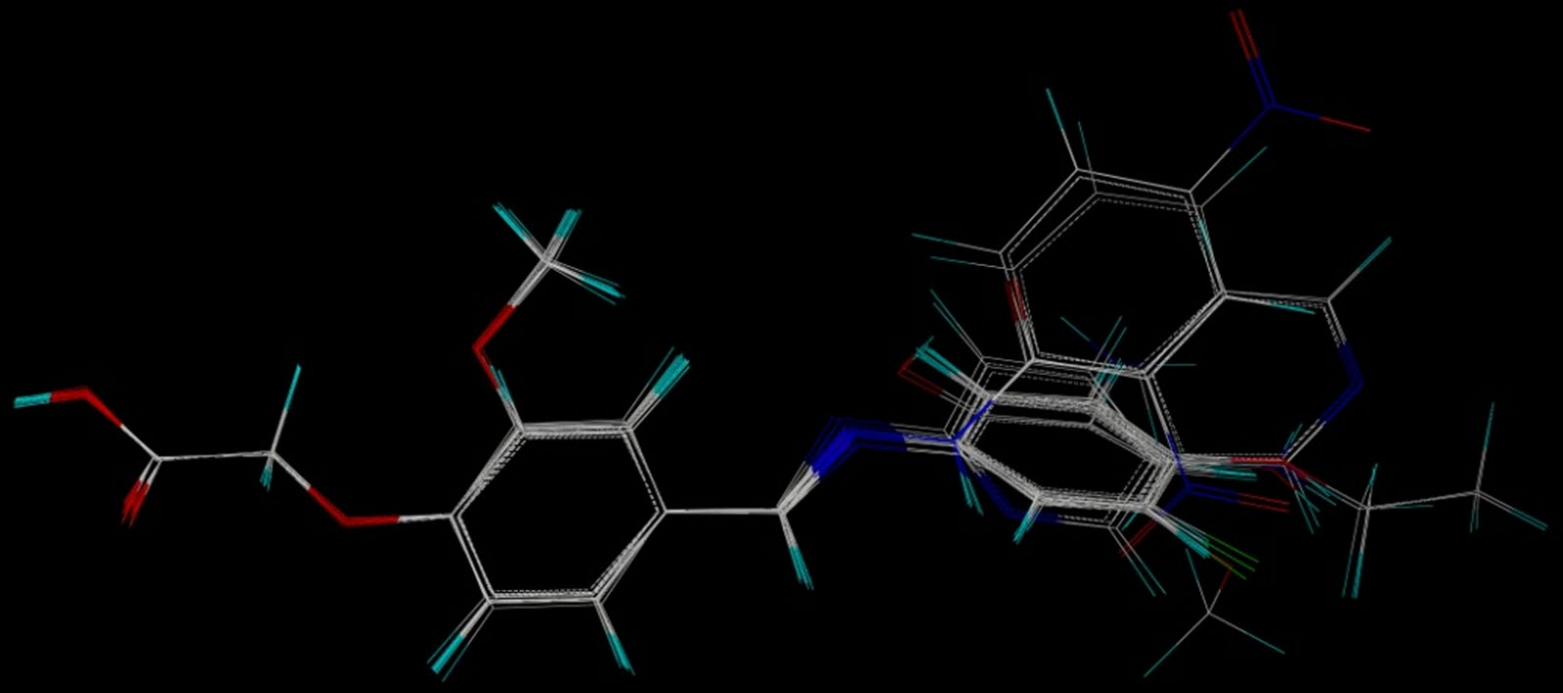
Training set molecules after alignment by field fit method

All the molecules were analysed for conformation and aligned by field fit method against the common substructure of all the molecules of training set (Fig. 8).

**Fig. 9:**
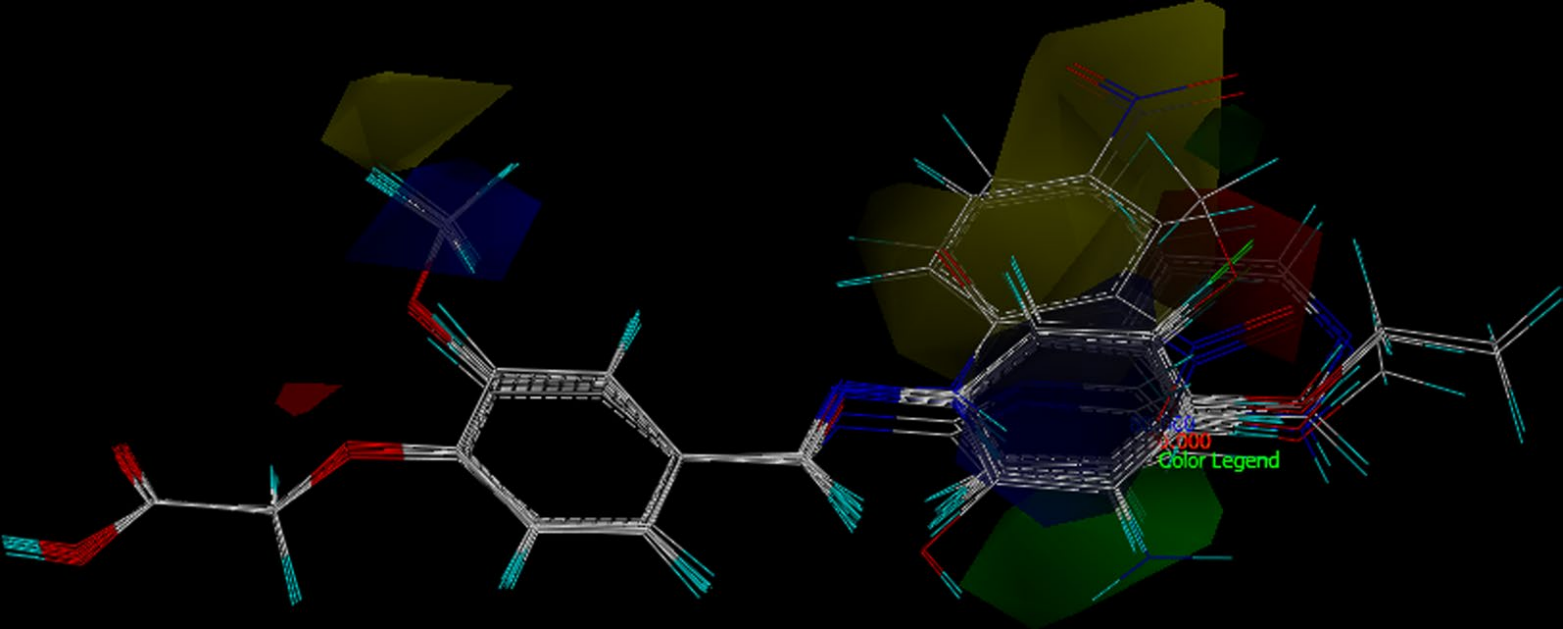
CoMSIA steric and electrostatic SD x coefficient contour plot; green contours indicate regions where steric bulk is favorable and yellow contours indicate regions where steric bulk is disfavored. Whereas Blue contours indicate regions where electronegative groups increase activity and red contours indicate regions where electropositive groups increase activity

**Fig. 10:**
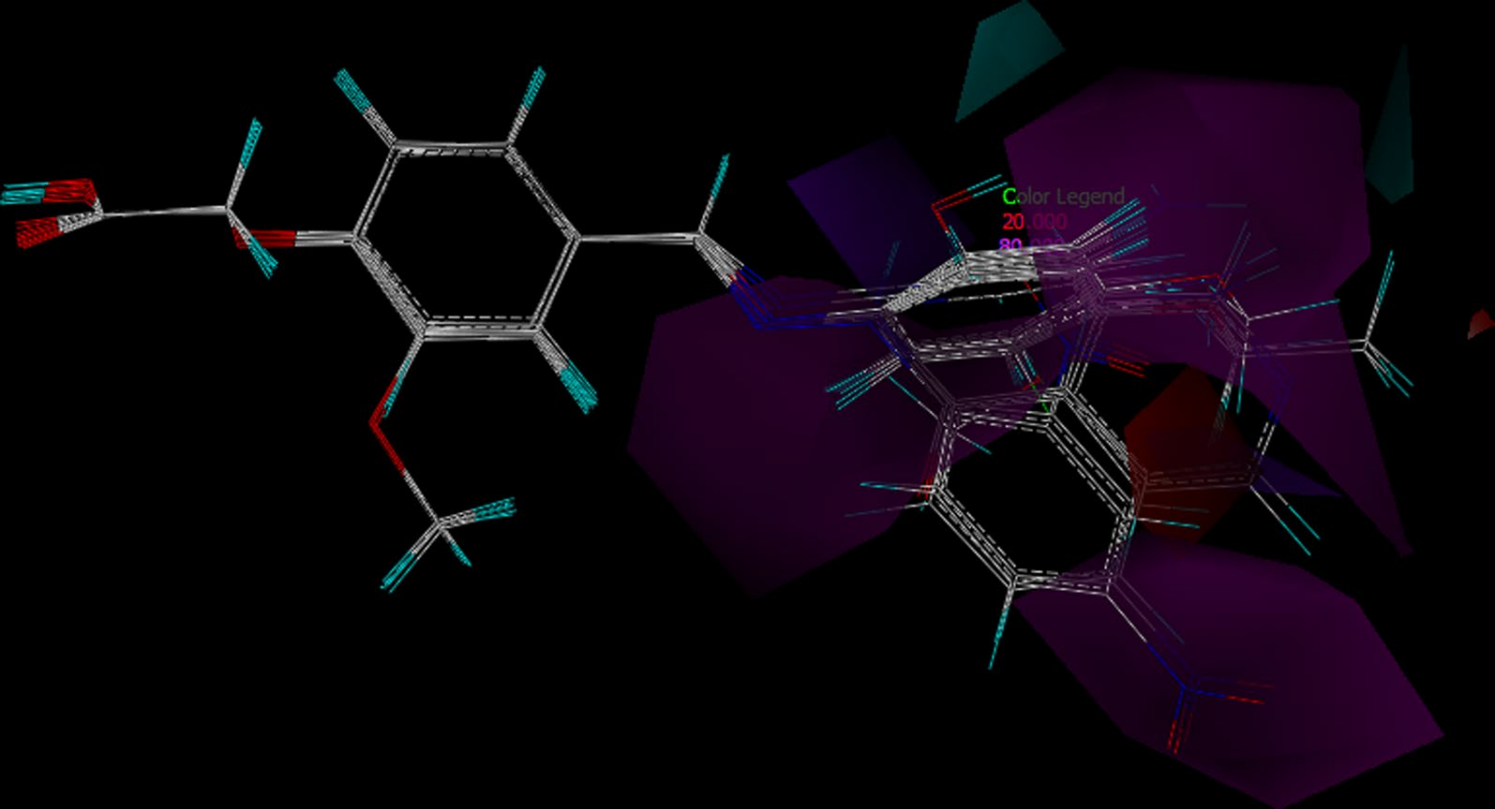
CoMSIA hydrogen bond donor and hydrogen bond acceptor SD × coefficient contour plot; cyan contours indicate regions where hydrogen bond donor increase activity and violet contours indicate regions where hydrogen bond donor decrease activity, whereas magenta contours indicate regions where hydrogen bond acceptors increase activity and red contours indicate regions where hydrogen bond donors decrease activity

**Fig. 11:**
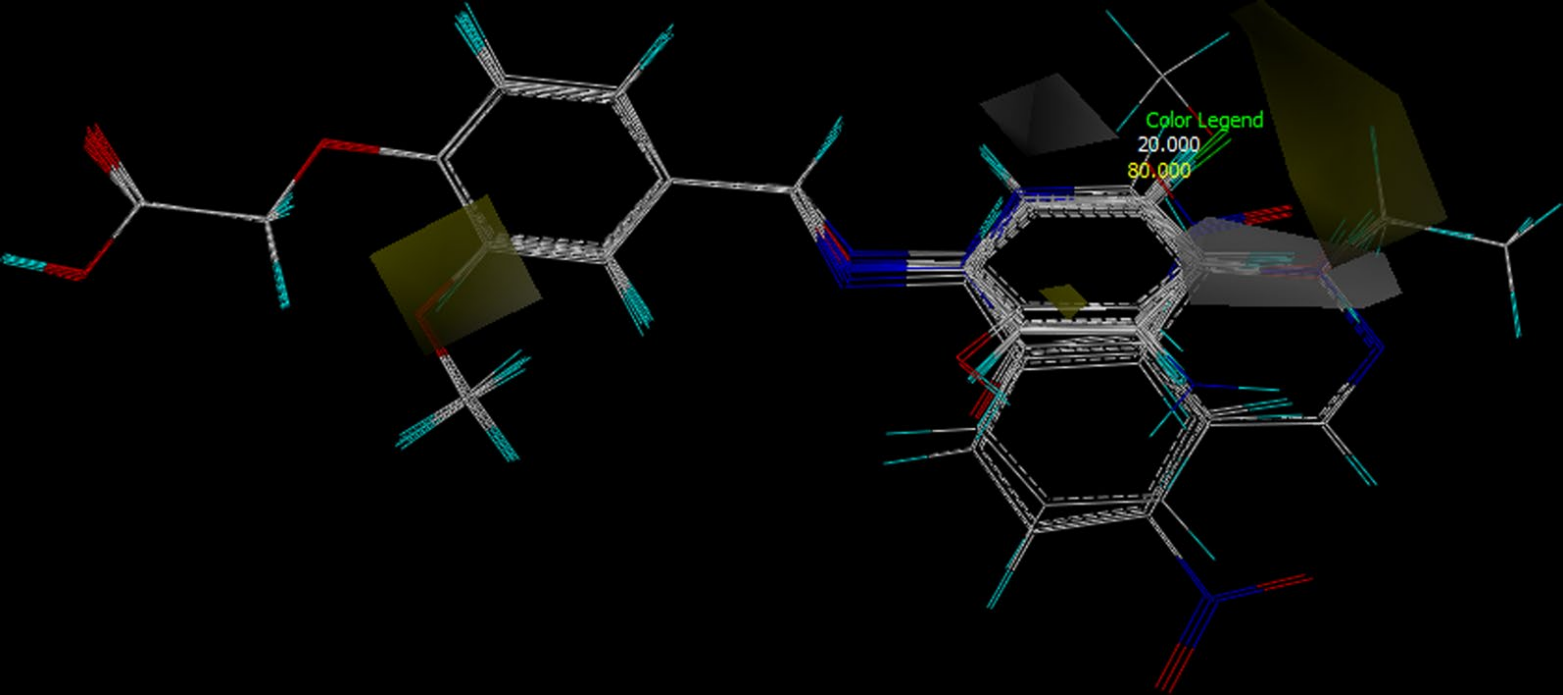
CoMSIA hydrophobic SD × coefficient contour plot; yellow contours indicate regions where hydrophobicity favors and white contours indicate regions where hydrophobicity disfavours

For CoMSIA, the use of molecular similarity indices avoids the arbitrary definitions of cut off values, and the descriptors can be calculated in all grid points [43]. Developed CoMSIA model provided better-defined contour maps and are as shown in Figs. 9, 10, 11.

### Structure–activity relationship for Glucose uptake activity

The structure–activity relationships based on the above CoMSIA contour maps are as follows; the basic moiety of phenoxyacetic acid with Schiff base linkage is important for possessing the activity as it is the common substructure in all the glitazones. The acidic phenoxyacetic acid, the aromatic ring and lipophillic imine linkage to the aromatic tail, all constitute the pharmacophoric features of the reported glitazones. The image depicted in Fig. 9 is combined contour of steric and electrostatic features for glucose uptake activity, where green and yellow contour area represents steric bulk favoured and disfavoured zone, respectively. Green contour map near to meta position of lipophilic aromatic ring indicates that steric bulk is favoured in that area and the same is observed in** CPD14, 15, 19** and** 20**. Yellow contour near methoxy group near to para position of aromatic ring indicates disfavoured zone for steric bulk and the same is observed in CPD06, 09, 10 and 17. Blue electrostatic contour indicates the favourable zone for electronegative groups and it appear near the methoxyl group in aromatic ring as evidence to it** CPD14, 19, 20, 21** and** 24** had similar substitution. In contrast, red contour from meta to para position of terminal aromatic ring indicates disfavoured zone for electronegative atoms and it was observed in** CPD10, 15, 17** and** 23** (Tables 7 and 8).

**Table 7:** Compounds considered for the training set

Compound	R′	Glucose uptake activity		
		lnGU	Predicted activity	Residual activity
CPD02		3.91	3.87	0.04
CPD03		4.39	4.36	0.03
CPD04		4.30	4.26	0.04
CPD05		4.20	4.15	0.05
CPD07		4.48	4.45	0.03
CPD08		4.50	4.40	0.10
CPD09		2.99	2.86	0.13
CPD10		3.73	3.64	0.09
CPD11		4.31	4.28	0.03
CPD12		4.27	4.23	0.04
CPD14		4.25	4.18	0.07
CPD15		3.81	3.78	0.03
CPD16		4.00	3.98	0.02
CPD17		3.56	3.49	0.07
CPD18		4.17	4.12	0.05
CPD19		4.50	4.48	0.02
CPD20		4.38	4.32	0.06
CPD21		4.44	4.45	0.01
CPD22		4.01	4.12	− 0.11
CPD23		3.69	3.61	0.08

**Table 8:** Compounds of the test set

Compound	R′	Glucose uptake activity		
		lnGU	Predicted activity	Residual activity
CPD01		3.80	3.74	0.06
CPD06		3.80	3.79	0.01
CPD13		3.22	3.16	0.06
CPD24		4.17	4.18	− 0.01

The combined contour of hydrogen bond donor and acceptor is shown in Fig. 10 where cyan and violet represent hydrogen bond donor favored and disfavored zones, whereas magenta and red represent hydrogen bond acceptor favored and disfavored zones, respectively.

Contour map for hydrophobicity is as depicted in Fig. 11. Where yellow and white colors are representing hydrophobicity favor and disfavor zones, respectively. Yellow region near methoxy of trunk aromatic group and some portion of tail aromatic group favors hydrophobicity, whereas white contour near para of tail aromatic group disfavors hydrophobicity.

## Experimental

### Pharmacophore modelling and database screening

Structure based pharmacophore protocol was adopted starting from the PPARγ protein–ligand complex and UNITY search was performed. For building the QUERY and running the UNITY search, we used the SYBYL X 2.1.1 software package (Tripose USA); the overall procedure includes pharmacophoric query generation and unity database search. To start the pharmacophore based virtual screening, we build the query by considering the conformation of Pioglitazone complexed with PPARγ (3CS8) protein, by taking the characteristic features of bound ligand (Pioglitazone) in complex with macromolecule (PPARγ). The special characters included to build the QUERY were, Hydrogen bond donor, Hydrogen bond acceptor, Hydrophobic and Aromatic feature from both ligand and macromolecule, with appropriate tolerance value. The program UNITY SEARCH performs the search algorithm for database of molecules against build query of pharmacophoric features, where the database of active molecules was searched against the build QUERY.

### Docking studies

The designed compounds were virtually sketched and the Docking operation was performed by using SERFLEX-DOCK program as a part of the SYBYL-X 2.1.1 software package (Tripose USA) and necessary calculations were done then after. The overall procedure of DOCKING program includes ligand preparation, protein preparation, protomol generation and docking of ligands.

Adaptation of Docking protocol and validation of the same were performed by comparing the binding poses of co-crystalized ligand with amino acids of target proteins PPARγ (pdb: 3CS8) before and after the docking study. All the designed and synthesized compounds along with pioglitazone were sketched in the molecular area provided with SYBYL-X 2.1.1 software package, prepared and stored, using ligand preparation wizard, where they were converted to their 3D form, energy minimized and charges were added. The force field used for energy minimization was MMFF94s [44] and charges added to each molecule using Gasteiger–Huckel method [45, 46]. The proteins from Protein Data Bank were prepared by adding Hydrogens and missing amino acids, deleting water molecules and finally applying charges. The energy minimization step was performed using MMFF94s as Force Field and applied Gasteiger–Marsili charges [47] with 1000 iterations of conjugate gradient method in convergence criterion of 1.0 kcal/mol. All these steps were performed on SYBYL-X 2.1.1 software platform using Biopolymer Preparation wizard. The active site of any target protein was identified by the Protomol generation step. In other words, Protomol is the ensemble of different amino acids of any target protein where the desired ligands will bind by different intermolecular interactions with the amino acids. In the present work we had generated the protomol by considering the binding mode of co-crystallized ligand inside the protein cavity. Finally, the prepared ligand and protein were interacted by GEOM mode, where 20 conformers of each ligand were generated, the stable conformer interacting with protein was identified and respective docking scores were then obtained as Total Score, Crash Score and Polar Score.

### Molecular dynamics simulation studies

Molecular Dynamics (MD) simulations were performed for the native protein (PPARγ) and their docked complexes of PPARγ-ligand using GROMACS version 4.5.5 [48] implemented with CHARMM 27 forcefield [49]. The topology file for all the ligands were generated by SwissParam using CHARMM all atoms forcefield [50]. The Van der Waal interactions were calculated with a distance cut-off of 1.0 nm. Partial Mesh Ewald (PME) summation was applied for long range electrostatics with 1 nm cut-off for columbic interaction [51]. Counter ions were included for the requirement of electro-neutrality condition of the system. The system was solvated using TIP3P water model [52, 53] and simulated in a triclinic box with protein atoms apart to 1.5 nm from the wall of the box dimensions along with periodic boundary conditions. The structures were first energy minimized using steepest descent algorithm with a tolerance of 1000 KJ/mol/nm. The system was equilibrated by applying position restrains on the complex and performing simulations using canonical NVT ensembles followed by NPT. Both the equilibration was run for 200 ps each at a temperature of 300 K and 1 bar. Temperature coupling was performed using velocity rescaling [54] with a coupling constant of 0.1 ps and the initial velocities were generated according to Maxwell distribution. Temperature–pressure coupling was performed using extended ensemble Parrinello–Rahman algorithm [55] with a coupling constant of 2 ps. The equilibrated system was then subjected to 5 ns of production run. A time step integration of 2 fs was used. The trajectories were saved every 500 steps and analyzed using GROMACS analysis tools and XMGRACE-5.1.22 program (http://plasma-gate.weizmann.ac.il/Grace/).

### Chemistry

All the synthetic work was done by procuring available laboratory grade reagents and analytical grade solvents. The solvents and reagents were used as such provided by the manufacturer. TLC was performed to monitor the reactions and to determine the purity of the products. All the reported compounds were purified by column chromatography. The melting points of the synthesized compounds were determined in open capillary method, expressed in  °C. IR spectra of compounds were recorded on Shimadzu FT-IR 8400-S spectrophotometer by KBr pellet technique and are expressed in cm^−1^. ^1^H-NMR and ^13^C-NMR spectra were recorded on Bruker 400 MHz FT-NMR spectrophotometer using DMSO D_6_ and CDCl_3_ as the solvents and TMS as internal standard (δ ppm). Mass spectra were obtained using LC–MS ACQUITY UPLC mass spectrometer under ES ionization at 70 eV and Time of flight detector. Retention Time (RT) also observed on the same UPLC instrument by maintaining the below mentioned optimized chromatographic condition: Column: C_18_ 1.7 micron, Flow rate: 0.4 ml/min, Run time: 15 min, Injection volume: 10 µl, Detector: PDA Detector, TOF, Elution: Gradient, Mobile phase: 0.1% FA in water and Acetonitrile, Column temperature: 60 °C.

### General procedure for the synthesis of sodium phenoxide

Sodium phenoxide from the vanillin and *p*-hydroxybenzaldehyde was prepared by taking 0.02 M equivalents of vanillin or *p*-hydroxybenzaldehyde in a beaker, added 0.02 M Sodium hydroxide and 20 ml of distilled water and the solution was mechanically stirred at room temperature, until whole solution became clear [56].

### General procedure for the synthesis of formylphenoxyacetic acid

Formylphenoxyacetic acid was prepared by modifying Williamsons ether synthesis protocol [28], by taking sodium phenoxide and treating it with equivalent amounts of chloroacetic acid in presence of sodium hydroxide. The whole solution of prepared sodium phenoxide in the previous step was taken in a beaker, to that 30 ml of chloroform was added and the solution was stirred mechanically for a period of 10 min at room temperature. To the above solution 0.02 M chloroacetic acid crystals dissolved in 15 ml of distilled water and another 0.02 M sodium hydroxide pellets dissolved in 15 ml of distilled water were also added. The whole solution was continued to stir for another 10 min. The resultant mixture was allowed to settle, then the whole mixture was poured into a separating funnel and the aqueous layer was taken in a round bottom flask, and the solution was refluxed with stirring at a temperature of 120–140 °C for 3 h. Reaction mixture was allowed to cool at room temperature and concentrated HCl was added drop wise until the precipitation stops, filtered and added 20 ml of chloroform and suspended in separating funnel. To this mixture saturated solution of sodium bicarbonate was added until whole precipitate goes into the aqueous phase. The aqueous phase thus separated was added concentrated HCl slowly, precipitate starts reappearing, filtered and dried.

*2*-*(4*-*formylphenoxy)acetic acid* (**CPD01**). Pale brown solid, yield 75%, mp 185–187 °C; IR (KBr, cm^−1^): 3448.84 (O–H, Acid), 3069.00 (ArC-H), 1759.14 (C=O, Acid), 1651.12 (C=O, Aldehyde), 1427.37 (C–C), 1226.77 (C–O);^1^H NMR (δ ppm, CDCl_3_): 4.487 (s, 2H, CH_2_), 6.837–7.655 (m, 4H, ArH), 9.694 (s, 1H, CHO); ^13^C NMR: (δ ppm, CDCl_3_): 55.734, 114.826, 114.956, 129.924, 132.351, 158.067, 163.321, 185.231; MS (m/z): M + 1 peak found 181.0835, (M + 1 peak calculated 181.16). Mass fragments (m/z): 181.0835, 182.0845; HPLC (RT): 3.19 min.

*2*-*(4*-*formyl*-*2*-*methoxyphenoxy)acetic acid* (**CPD02**). Off white solid, yield 60%, mp 145–147 °C; IR (KBr, cm^−1^): 3510.56 (O–H, Acid), 3091.99 (ArC-H), 1766.85 (C=O, Acid), 1643.41 (C=O, Aldehyde), 1411.94 (C–C), 1273.06 (C–O);^1^H NMR (δ ppm, CDCl_3_): 3.849 (s, 3H, OCH_3_), 4.656 (s, 2H, CH_2_), 6.814–7.347 (m, 3H, ArH), 9.758 (s, 1H, CHO);^13^C NMR: (δ ppm, CDCl_3_): 52.315, 54.125, 55.734, 108.950, 109.521, 124.021, 130.328, 150.356, 153.745, 163.351, 185.213; MS (m/z): M + 1 peak found 211.0897, (M + 1 peak calculated 211.05). Mass fragments (m/z): 211.0897, 212.0941; HPLC (RT): 3.33 min.

### General procedure for the synthesis of Schiff base

The imine (–CH=N–) linkage between an amine and aldehyde was made by taking equivalent quantity of amine and aldehyde with catalytic amount of glacial acetic acid, stirred with or without heating according to the procedure reported by *Hugo Schiff* (1864) [29]. In the present study, we have synthesized the Schiff base by taking equimolar quantities of formylphenoxyacetic acid and substituted aromatic amine. Both of them were dissolved in absolute ethanol and mixed together, to that few drops of glacial acetic acid and few activated molecular sieves were added and finally stirred and refluxed at 80–90 °C for 8–12 h. The reaction was monitored from time to time by drawing samples and checked TLC. The formed precipitate was filtered washed with minimal quantities of cold aqueous ethanol and purified by column chromatography using 25% ethyl acetate in pet ether as mobile phase and silica gel as stationary phase.

*2*-*(4*-*((E)*-*(phenylimino)methyl)phenoxy)acetic acid* (**CPD03**). Yellow amorphous solid, yield 73%, mp 193–195 °C; IR (KBr, cm^−1^): 3263.66 (O–H, Acid), 3016.77 (Ar–H), 1743.71 (C=O, Acid), 1604.83 (C=N, Imine), 1427.37 (C–N), 1273.06 (C–O);^1^H NMR (δ ppm, CDCl_3_): 4.574 (s, 2H, CH_2_), 6.548–7.478 (m, 9H, ArH), 8.255 (s, 1H, CH=N);^13^C NMR: (δ ppm, CDCl_3_): 65.054, 114.410, 114.410, 122.312, 122.312, 126.124, 127.321, 130.121, 130.321, 130.245, 130.245, 153.278, 160.145, 163.004, 173.258; MS (m/z): M + 1 peak found 256.1238, (M + 1 peak calculated 256.09). Mass fragments (m/z): 256.1238, 257.1389; HPLC (RT): 4.15 min.

*2*-*(4*-*((E)*-*(4*-*methoxyphenylimino)methyl)phenoxy)acetic acid* (**CPD04**). Pale yellow amorphous solid, yield 68%, mp 189–192 °C;IR (KBr, cm^−1^): 3425.69 (O–H, Acid), 3070.78 (Ar–H), 1766.85 (C=O, Acid), 1604.83 (C=N, Imine), 1288.49 (C–N), 1249.91 (C–O);^1^H NMR (δ ppm, DMSO D_6_): 3.737 (s, 3H, OCH_3_), 4.653 (s, 2H, CH_2_), 6.915–7.811 (m, 8H, ArH), 8.510 (s, 1H, CH=N); ^13^C NMR: (δ ppm, DMSO D_6_): 52.296, 55.734, 65.106, 114.826, 114.955, 115.183, 115.236, 122.620, 122.643, 129.958, 130.338, 130.429, 130.482, 144.870, 144.931, 157.983, 158.044, 158.067, 158.112, 160.305, 160.715, 169.381; MS (m/z): M + 1 peak found 286.2908, (M + 1 peak calculated 286.10). Mass fragments (m/z); 285.2146, 286.2908, 287.0124; HPLC (RT): 2.17 min.

*2*-*(4*-*((E)*-*N*-*(4*-*hydroxyphenyl)carboximidoyl)phenoxy)acetic acid* (**CPD05**). Yellowamorphous solid, yield 69%.mp 195–197 °C; IR (KBr, cm^−1^): 3549.14 (O–H, Phenol), 3394.83 (O–H, Acid), 3070.78 (Ar–H), 1694.45 (C=O, Acid), 1604.83 (C=N, Imine), 1342.50 (C–N), 1273.06 (C–O);^1^H NMR (δ ppm, DMSO D_6_): 4.432 (s, 2H, CH_2_), 6.620–7.607 (m, 8H, ArH), 8.187 (s, 1H, CH=N);^13^C NMR: (δ ppm, DMSO D_6_): 65.045, 77.172, 77.490, 77.695, 77.817, 114.743, 114.933, 115.912, 116.018, 116.473, 122.005, 129.973, 131.795, 160.657, 190.569; MS (m/z): M + 1 peak found 272.1455, (M + 1 peak calculated 272.08). Mass fragments (m/z): 272.1455, 266.9080, 259.1119; HPLC (RT): 3.14 min.

*2*-*(4*-*((E)*-*(pyridin*-*2*-*ylimino)methyl)phenoxy)acetic acid* (**CPD06**). Pale brown amorphous solid, yield 71%, mp 188–190 °C; IR (KBr, cm^−1^): 3356.25 (O–H, Acid), 3070.78 (Ar–H), 1751.42 (C=O, Acid), 1589.40 (C=N, Imine), 1512.24 (C=N, Pyridine), 1319.35 (C–N), 1226.77 (C–O);^1^H NMR (δ ppm, CDCl_3_): 4.738 (s, 2H, CH_2_), 6.991–7.851 (m, 8H, ArH), 8.956 (s, 1H, CH=N);^13^C NMR: (δ ppm, CDCl_3_): 65.878, 114.401, 114.401, 116.212, 122.412, 126.124, 130.235, 130.201, 137.332, 150.412, 160.114, 160.718, 163.025, 173.280; MS (m/z): M + 1 peak found 257.1179, (M + 1 peak calculated 257.08). Mass fragments (m/z): 257.1179, 255.1874, 250.9812; HPLC (RT): 6.05 min.

*2*-*(4*-*((E)*-*(3*-*chlorophenylimino)methyl)phenoxy)acetic acid* (**CPD07**). Dark yellow amorphous solid, yield 70%, mp 184–186 °C; IR (KBr, cm^−1^): 3441.12 (O–H, Acid), 3070.78 (Ar–H), 1697.41 (C=O, Acid), 1604.83 (C=N, Imine), 1381.08 (C–N), 1280.78 (C–O), 732.97 (C–Cl);^1^H NMR (δ ppm, CDCl_3_): 4.600 (s, 2H, CH_2_), 6.564–7.478 (m, 8H, ArH), 8.255 (s, 1H, CH=N); ^13^C NMR: (δ ppm, CDCl_3_): 67.624, 114.410, 114.410, 120.452, 122.621, 126.110, 127.458, 130.221, 130.221, 131.252, 135.694, 154.623, 160.121, 163.021, 173.035; MS (m/z): M + 1 peak found 290.0435, (M + 1 peak calculated 290.05). Mass fragments (m/z): 290.0435, 288.0449; HPLC (RT): 12.81 min.

*2*-*(4*-*((E)*-*N*-*(5*-*chloro*-*2*-*hydroxyphenyl)carboximidoyl)phenoxy)acetic acid* (**CPD08**). Pale brown amorphous solid, yield 80%, mp 201–202 °C; IR (KBr, cm^−1^): 3394.83 (O–H, Phenolic), 3063.06 (Ar–H), 1751.42 (C=O, Acid), 1589.40 (C=N, Imine), 1381.08 (C–N), 1226.77 (C–O), 678.97 (C–Cl);^1^H NMR (δ ppm, DMSO D_6_): 4.795 (s, 2H, CH_2_), 6.854–7.949 (m, 7H, ArH), 8.596 (s, 1H, CH=N);^13^C NMR: (δ ppm, DMSO D_6_): 52.303, 52.357, 65.060, 65.113, 65.174, 115.107, 115.160, 115.456, 115.509, 115.972, 117.649, 119.220, 123.197, 123.318, 126.566, 129.837, 130.489, 131.294, 132.113, 139.854, 150.478, 160.533, 160.981, 163.121, 169.988, 170.231, 191.707; MS (m/z): M + 1 peak found 306.5956, (M + 1 peak calculated 306.05). Mass fragments (m/z): 306.5956, 305.5344, 305.8690; HPLC (RT): 12.88 min.

*2*-*(4*-*((1E)*-*(2*-*(2,4*-*dinitrophenyl)hydrazin*-*1*-*ylidene)methyl)phenoxy)acetic acid* (**CPD09**). Orange amorphous solid, yield 82%, mp 193–195 °C; IR (KBr, cm^−1^): 3618.58 (N–H), 3294.53 (O–H, Acid), 3109.35 (Ar–H), 1728.28 (C=O, Acid), 1604.83 (C=N, Imine), 1504.53 (NO_2_), 1334.78 (C–N), 1257.63 (C–O);^1^H NMR (δ ppm, DMSO D_6_): 4.552 (s, 2H, CH_2_), 6.870–8.241 (m, 7H, ArH), 8.962 (s, 1H, CH=N), 11.327 (s, 1H, NH);^13^C NMR: (δ ppm, DMSO D_6_): 65.037, 77.460, 77.786, 77.983, 78.105, 115.047, 116.891, 123.311, 126.983, 129.108, 129.268, 129.662, 144.916, 148.550, 160.062, 173.155; MS (m/z): M + 1 peak found 361.1061, (M + 1 peak calculated 361.07). Mass fragments (m/z): 361.1061, 351.1051, 349.2133; HPLC (RT): 8.56 min.

*2*-*(4*-*((1E)*-*((pyridin*-*4*-*ylformamido)imino)methyl)phenoxy)acetic acid* (**CPD10**). White amorphous solid, yield 80%, mp 200–202 °C; IR (KBr, cm^−1^): 3572.29 (N–H), 3394.83 (O–H, Acid), 3039.91 (Ar–H), 1743.71 (C=O, Acid), 1666.55 (C=O, Amide), 1604.83 (C=N, Imine), 1296.21 (C–N), 1249.91 (C–O);^1^HNMR (δ ppm, CDCl_3_): 4.533 (s, 2H, CH_2_), 6.831–8.304 (m, 8H, ArH), 8.653 (s, 1H, CH=N), 11.547 (s, 1H, NH);^13^C NMR: (δ ppm, CDCl_3_): 67.037, 77.780, 77.786, 77.883, 77.905, 126.311, 129.983, 140.108, 143.268, 149.662, 149.916, 160.050, 160.062, 173.155;MS (m/z): M + 1 peak found 300.1331, (M + 1 peak calculated 300.09). Mass fragments (m/z): 300.1331, 298.0772; HPLC (RT): 2.95 min.

*2*-*(4*-*((E)*-*(4*-*ethoxyphenylimino)methyl)phenoxy)acetic acid* (**CPD11**). Off white amorphous solid, yield 81%, mp 186–188 °C; IR (KBr, cm^−1^): 3394.83 (O–H, Acid), 3070.78 (Ar–H), 1651.12 (C=O, Acid), 1604.83 (C=N, Imine), 1350.22 (C–N), 1242.20 (C–O);^1^H NMR (δ ppm, DMSO D_6_): 1.288 (t, 3H, CH_3_), 3.918 (q, 2H, CH_2_), 4.567 (s, 2H, CH_2_), 6.474–7.736 (m, 8H, ArH), 8.333 (s, 1H, CH=N);^13^C NMR: (δ ppm, DMSO D_6_): 63.590, 63.891, 65.098, 78.044, 78.371, 78.368, 78.697, 114.826, 115.001, 115.623, 115.876, 122.104, 130.156, 144.749, 157.217, 157.346; MS (m/z): M + 1 peak found 300.2240, (M + 1 peak calculated 300.12). Mass fragments (m/z): 300.2240, 299.2101, 297.0445; HPLC (RT): 7.43 min.

*2*-*(4*-*((E)*-*(4*-*aminophenylimino)methyl)phenoxy)acetic acid* (**CPD12**). White amorphous solid, yield 80%, mp 196–198 °C; IR (KBr, cm^−1^): 3502.85 (N–H, Amine), 3394.83 (O–H, Acid), 3017.23 (Ar–H), 1651.20 (C=O, Acid), 1604.83 (C=N, Imine), 1330.51 (C–N), 1242.20 (C–O);^1^H NMR (δ ppm, CDCl_3_): 4.642 (s, 2H, CH_2_), 6.545–8.039 (m, 8H, ArH), 8.379 (s, 1H, CH=N);^13^C NMR: (δ ppm, CDCl_3_): 67.124, 77.784, 77.784, 79.883, 79.905, 125.311, 125.383, 129.108, 136.268, 145.662, 146.916, 160.140, 163.062, 173.051; MS (m/z): M + 1 peak found 271.1371, (M + 1 peak calculated 271.10). Mass fragments (m/z): 271.1371, 270.9644, 270.1306; HPLC (RT): 3.06 min.

*2*-*(2*-*methoxy*-*4*-*((E)*-*(phenylimino)methyl)phenoxy)acetic acid* (**CPD13**). Yellow amorphous solid, yield 79%, mp 194–196 °C; IR (KBr, cm^−1^): 3309.96 (OH, Acid), 3024.48 (ArH), 1675.20 (C=O, Acid), 1597.11 (C=N, Imine), 1265.35 (C–N), 1226.77 (C–O);^1^H NMR (δ ppm, CDCl_3_): 3.796 (s, 3H, OCH_3_), 4.496 (s, 2H, CH_2_), 6.635–7.598 (m, 8H, ArH), 9.050 (s, 1H, CH=N);^13^C NMR: (δ ppm, CDCl_3_): 56.285, 67.912, 82.354, 83.381, 89.878, 89.999, 111.326, 121.664, 127.128, 132.311, 133.950, 145.422, 149.313, 150.621, 160.681, 174.838; MS (m/z): M + 1 peak found 286.1418, (M + 1 peak calculated 286.29). Mass fragments (m/z): 286.1418, 263.1089, 233.0761; HPLC (RT): 9.51 min.

*2*-*(4*-*((E)*-*(4*-*methoxyphenylimino)methyl)*-*2*-*methoxyphenoxy)acetic acid* (**CPD14**). Yellow amorphous solid, yield 75%, mp 193–195 °C; IR (KBr, cm^−1^): 3394.83 (O–H, Acid), 3070.78 (Ar–H), 1666.55 (C=O, Acid), 1597.11 (C=N, Imine), 1275.35 (C–N), 1226.20 (C–O);^1^H NMR (δ ppm, CDCl_3_): 3.775 (s, 3H, OCH_3_), 3.940 (s, 3H, OCH_3_), 4.782 (s, 2H, CH_2_), 6.657–7.607 (m, 7H, ArH), 8.371 (s, 1H, CH=N);^13^C NMR: (δ ppm, CDCl_3_): 55.885, 56.220, 67.856, 82.364, 83.364, 84.578, 84.580, 122.664, 124.128, 124.221, 130.311, 143.950, 145.422, 149.313, 158.621, 160.681, 174.838; MS (m/z): M + 1 peak found 317.1613, (M + 1 peak calculated 316.11). Mass fragments (m/z): 317.1613, 316.1502, 315.0157; HPLC (RT): 6.51 minY.

*2*-*(4*-*((E)*-*N*-*(4*-*hydroxyphenyl)carboximidoyl)*-*2*-*methoxyphenoxy)acetic acid* (**CPD15**). Yellowish orange amorphous solid, yield 85%, mp 198–200 °C; IR (KBr, cm^−1^): 3410.26 (O–H, Phenol), 3178.79 (O–H, Acid), 3078.49 (Ar–H), 1666.55 (C=O, Acid), 1597.11 (C=N, Imine), 1273.06 (C–N), 1219.05 (C–O);^1^H NMR (δ ppm, CDCl_3_): 3.849 (s, 3H, OCH_3_), 4.587 (s, 2H, CH_2_), 6.710–8.282 (m, 7H, ArH), 8.884 (s, 1H, CH=N);^13^C NMR: (δ ppm, CDCl_3_): 56.212, 67.895, 114.364, 115.364, 116.578, 116.580, 122.564, 125.128, 126.221, 130.311, 130.350, 149.422, 152.313, 160.621, 164.681, 173.938; MS (m/z): M + 1 peak found 301.0727, (M + 1 peak calculated 302.10). Mass fragments (m/z): 301.0727, 300.0725; HPLC (RT): 10.86 min.

*2*-*(2*-*methoxy*-*4*-*((E)*-*(pyridin*-*2*-*ylimino)methyl)phenoxy)acetic acid* (**CPD16**). White amorphous solid, yield 80%, mp 187–189 °C; IR (KBr, cm^−1^): 3425.69 (O–H, Acid), 3078.20 (Ar–H), 1690.55 (C=O, Acid), 1604.83 (C=N, Imine), 1273.06 (C–N), 1218.05 (C–O);^1^H NMR (δ ppm, CDCl_3_): 3.817 (s, 3H, OCH_3_), 4.404 (s, 2H, CH_2_), 6.414–7.829 (m, 7H, ArH), 8.884 (s, 1H, CH=N);^13^C NMR: (δ ppm, CDCl_3_): 56.202, 67.915, 114.464, 115.384, 116.618, 122.464, 122.521, 127.128, 137.311, 149.922, 150.413, 152.621, 160.181, 160.712, 173.895;MS (m/z): M + 1 peak found 287.4666, (M + 1 peak calculated 287.10). Mass fragments (m/z): 287.4666, 278.4613, 271.9437; HPLC (RT): 0.50 min.

*2*-*(4*-*((E)*-*N*-*(5*-*chloro*-*2*-*hydroxyphenyl)carboximidoyl)*-*2*-*methoxyphenoxy)acetic acid* (**CPD17**). Pale Yellow crystalline solid, yield 80%, mp 193–195 °C; IR (KBr, cm^−1^): 3525.99 (O–H, Phenol), 3394.83 (O–H, Acid), 3078.20 (Ar–H), 1650.12 (C=O, Acid), 1604.83 (C=N, Imine), 1265.35 (C–N), 1226.77 (C–O), 709.83 (C–Cl);^1^H NMR (δ ppm, CDCl_3_): 3.827 (s, 3H, OCH_3_), 4.443 (s, 2H, CH_2_), 6.771–7.571 (m, 6H, ArH), 8.357 (s, 1H, CH=N);^13^C NMR: (δ ppm, CDCl_3_): 56.212, 67.905, 114.489, 115.584, 117.518, 119.864, 122.525, 126.128, 127.311, 130.712, 132.652, 149.922, 152.113, 159.621, 164.612, 173.945; MS (m/z): M + 1 peak found 336.0919, (M + 1 peak calculated 336.06). Mass fragments (m/z): 336.0919, 335.0671, 334.0759. HPLC (RT): 7.12 min.

*2*-*(2*-*methoxy*-*4*-*((1E)*-*(2*-*phenylhydrazin*-*1*-*ylidene)methyl)phenoxy)acetic acid* (**CPD18**). White amorphous solid, yield 79%, mp 180–182 °C; IR (KBr, cm^−1^): 3502.85 (N–H), 3394.83 (O–H, Acid), 3032.20 (Ar–H), 1653.41 (C=O, Acid), 1597.11 (C=N, Imine), 1265.35 (C–N), 1225.77 (C–O);^1^H NMR (δ ppm, CDCl_3_): 3.809 (s, 3H, OCH_3_), 4.463 (s, 2H, CH_2_), 6.628–7.741 (m, 9H, ArH), 8.282 (s, 1H, CH=N);^13^C NMR: (δ ppm, CDCl_3_): 56.236, 67.865, 114.499, 115.484, 116.318, 116.321, 118.864, 122.515, 127.128, 129.611, 129.625, 143.023, 143.152, 149.922, 152.123, 173.845;MS (m/z): M + 1 peak found 301.1485, (M + 1 peak calculated 301.11). Mass fragments (m/z): 301.1485, 298.0772; HPLC (RT): 7.56 min.

*2*-*(4*-*((1E)*-*(2*-*(2,4*-*bis(hydroxynitroso)phenyl)hydrazin*-*1*-*ylidene)methyl)*-*2*-*methoxyphenoxy)acetic acid* (**CPD19**). Orange red amorphous solid, yield 79%, mp 189–192 °C; IR (KBr, cm^−1^): 3525.99 (N–H), 3279.10 (O–H, Acid), 3086.21 (Ar–H), 1749.41 (C=O, Acid), 1620.26 (C=N, Imine), 1504.53 (NO_2_), 1327.07 (C–N), 1219.05 (C–O);^1^H NMR (δ ppm, CDCl_3_): 3.865 (s, 3H, OCH_3_), 4.584 (s, 2H, CH_2_), 6.766–8.267 (m, 6H, ArH), 8.953 (s, 1H, CH=N), 11.369 (s, 1H, -NH);^13^C NMR: (δ ppm, CDCl_3_): 60.825, 76.622, 82.354, 82.681, 82.878, 82.999, 114.326, 118.097, 121.664, 127.128, 132.311, 133.950, 134.422, 142.313, 149.621, 153.681, 154.561, 154.758, 174.838; MS (m/z): M + 1 peak found 391.1217, (M + 1 peak calculated 391.08). Mass fragments (m/z): 391.1217, 349.2133; HPLC (RT): 8.42 min.

*2*-*(2*-*methoxy*-*4*-*((1E)*-*((pyridin*-*4*-*ylformamido)imino)methyl)phenoxy)acetic acid* (**CPD20**). Yellow amorphous solid, yield 71%, mp 184–186 °C; IR (KBr, cm^−1^): 3487.42 (N–H), 3240.52 (O–H, Acid), 3078.49 (Ar–H), 1743.71 (C=O, Amide), 1666.55 (C=O, Acid), 1604.83 (C=N, Imine), 1265.35 (C–N), 1226.77 (C–O);^1^H NMR (δ ppm, CDCl_3_): 3.820 (s, 3H, OCH_3_), 4.525 (s, 2H, CH_2_), 6.726–8.284 (m, 7H, ArH), 8.657 (s, 1H, CH=N), 11.662 (s, 1H, NH);^13^C NMR: (δ ppm, CDCl_3_): 56.212, 67.954, 114.421, 115.212, 122.545, 122.854, 122.865, 127.121, 140.912, 143.032, 149.832, 149.832, 149.954, 152.121, 163.423, 173.323; MS (m/z): M + 1 peak found 330.1415, (M + 1 peak calculated 330.10); HPLC (RT): 3.09 min.

*2*-*(4*-*((E)*-*(3*-*methoxyphenylimino)methyl)*-*2*-*methoxyphenoxy)acetic acid* (**CPD21**). Green amorphous solid, yield 70%, mp 238–240 °C; IR (KBr, cm^−1^): 3363.97 (O–H, Acid), 3063.06 (Ar–H), 1705.10 (C=O, Acid), 1604.83 (C=N, Imine), 1265.35 (C–N), 1211.34 (C–O);^1^H NMR (δ ppm, CDCl_3_): 3.524 (s, 3H, OCH_3_), 3.597 (s, 3H, OCH_3_), 4.450 (s, 2H, CH_2_), 6.377–7.625 (m, 8H, ArH), 8.357 (s, 1H, CH=N);^13^C NMR: (δ ppm, CDCl_3_): 55.912, 56.212, 67.954, 108.312, 112.875, 114.421, 115.412, 122.545, 127.185, 131.165, 149.912, 152.032, 154.232, 160.123, 162.023, 173.723;MS (m/z): M + 1 peak found 316.1502, (M + 1 peak calculated 316.11); HPLC (RT): 4.63 min.

*2*-*(4*-*((E)*-*(4*-*ethoxyphenylimino)methyl)*-*2*-*methoxyphenoxy)acetic acid* (**CPD22**). White amorphous solid, yield 80%, mp 197–199 °C; IR (KBr, cm^−1^): 3425.69 (O–H, Acid), 3070.78 (Ar–H), 1666.55 (C=O, Acid), 1597.11 (C=N, Imine), 1280.78 (C–N), 1257.63 (C–O);^1^H NMR (δ ppm, DMSO D_6_): 1.307 (t, 3H, CH_3_), 3.819 (s, 3H, OCH_3_), 3.918 (q, 2H, CH_2_), 4.569 (s, 2H, CH_2_), 6.784–7.776 (m, 7H, ArH), 8.303 (s, 1H, CH=N);^13^C NMR: (δ ppm, DMSO D_6_): 55.999, 63.656, 65.826, 110.342, 112.990, 115.335, 122.620, 123.599, 130.231, 144.764, 149.507, 150.546, 157.338, 158.228, 170.489, 172.386; MS (m/z): M + 1 peak found 330.1653, (M + 1 peak calculated 330.13). Mass fragments (m/z): 330.1653, 328.2463, 327.2336; HPLC (RT): 7.56 min.

*2*-*(4*-*((E)*-*(4*-*aminophenylimino)methyl)*-*2*-*methoxyphenoxy)acetic acid* (**CPD23**). Dark red amorphous solid, yield 80%, mp 201–203 °C; IR (KBr, cm^−1^): 3448.84 (N–H, Amine), 3340.82 (O–H, Acid), 3070.78 (Ar–H), 1666.55 (C=O, Acid), 1604.83 (C=N, Imine), 1280.78 (C–N), 1219.06 (C–O);^1^H NMR (δ ppm, CDCl_3_): 3.841 (s, 3H, OCH_3_), 4.644 (s, 2H, CH_2_), 6.564–7.674 (m, 7H, ArH), 8.352 (s, 1H, CH=N);^13^C NMR: (δ ppm, CDCl_3_): 56.212, 67.954, 114.412, 115.475, 117.621, 122.512, 123.145, 127.185, 143.265, 146.912, 149.932, 152.132, 160.189, 173.874; MS (m/z): M + 1 peak found 301.1713, (M + 1 peak calculated 301.11). Mass fragments (m/z): 301.1713, 299.2177; HPLC (RT): 10.83 min.

*2*-*(4*-*((E)*-*(3*-*aminophenylimino)methyl)*-*2*-*methoxyphenoxy)acetic acid* (**CPD24**). Dark yellow amorphous solid, yield 70%, mp 186–188 °C; IR (KBr, cm^−1^): 3394.83 (N–H, Amine), 3340.82 (O–H, Acid), 3055.35 (Ar–H), 1651.12 (C=O, Acid), 1589.40 (C=N, Imine), 1265.35 (C–N), 1219.05 (C–O);^1^H NMR (δ ppm, CDCl_3_): 3.820 (s, 3H, OCH_3_), 4.678 (s, 2H, CH_2_), 6.905–7.458 (m, 7H, ArH), 8.510 (s, 1H, CH=N);^13^C NMR: (δ ppm, CDCl_3_): 56.212, 67.954, 107.712, 112.375, 114.321, 114.812, 115.445, 122.585, 127.165, 130.912, 149.745, 149.932, 152.132, 160.189, 173.923;MS (m/z): M + 1 peak found 301.1485, (M + 1 peak calculated 301.11). Mass fragments (m/z): 301.1485, 299.2177, 283.2071; HPLC (RT): 0.70 min.

### Cytotoxic assay

Procedure adopted for cytotoxic assay was MTT assay by using 3T3-L1 cell lines. Tests were performed in 96-well plates. Cells were seeded (10,000 cells/well) and grown to maturation. Mature adipocytes were incubated with either 0.2% dimethylsulfoxide (DMSO) or the test samples (250–3.125 μg/ml) for 48 h. A cell viability assay was performed as per the manufacturer’s instructions. A total of 50 µl of the MTT solution (5 mg/ml) was added to each well and the plates were incubated for 3 h at 37 °C. After incubation, 200 µl of DMSO was added to the wells followed by gentle shaking to solubilise the formazan dye. Absorbance was read at 560 nm using a microplate reader to determine the formazan concentration, which is proportional to the number of live cells [36]. Surviving cell fraction was calculated according to the following equation:

$${\text{Cell viability }}\left( \% \right) \, = \, \left( {{\text{A}}_{\text{s}} /{\text{ A}}_{\text{c}} } \right) \times 100$$

where, A_s_ is the absorbance of the sample and A_c_ is the absorbance of the control, and the cytotoxicity is expressed as a percentage relative to control cells (without test sample supplementation).

### Glucose uptake assay

Monolayer of L-6 cells was maintained at sub confluent conditions in growth media containing DMEM with 4.5 g/l glucose, 100 U/ml penicillin, 100-µg/ml streptomycin, and 10% fetal bovine serum. Cells were maintained in a humidified 37 °C incubator with ambient oxygen and 5% CO_2_. Cells were maintained in continuous passage by trypsinization of sub confluent cultures using TPVG solution [38].

Cells were cultured on 24 well plates and incubated for 48 h at 37 °C in CO_2_ incubator. When semi confluent monolayer was formed the culture were renewed with serum free DMEM containing 0.2% Bovine serum albumin (BSA) and incubated for 18 h at 37 °C in CO_2_ incubator. After 18 h media was discarded and cells were washed with KRP buffer once. The cells were then treated with Insulin and test compounds. Consequently glucose solution (1 M) was added and incubated for half an hour. Supernatant was collected for glucose estimation and glucose uptake was terminated by washing the cells three times with 1 ml ice-cold KRP buffer. Subsequent freezing and thawing three times lysed all cells. Cell lysate was collected for glucose estimation [39, 40]. Glucose uptake was calculated as the difference between the initial and final glucose content in the incubated medium by GOD-POD method [57, 58]. 10 µl of sample was mixed with 1 ml of reagent (GOD-POD reagent) and incubated for 10 min at 37 °C. The absorbance of standard (A_standard_) and test compounds (A_sample_) against blank was measured at 505 nm. Formula is;

$${\text{Glucose concentration }}\left( {{\text{mg}}/{\text{dl}}} \right) \, = \, \left( {{\text{A}}_{\text{sample}} /{\text{A}}_{\text{standard}} } \right) \, \times { 1}00$$

## 3D-QSAR study

CoMSIA is a powerful and established tool for building 3D-QSAR models that can be applied to drug design [46]. The regular protocol for CoMSIA study and threedimensional structure building and all the modelling were carried out using the SYBYL-X 2.1.1 program package and the conformations of the compounds in the training and test sets were generated using the systematic conformational search method implemented in SYBYL-X 2.1.1. The molecules were drawn and analysed for conformation to ensure that all the molecules possessing same E configuration and with same scaffold arrangement. Energy minimization was affected using the MMFF94 s [44] with a distance dependent dielectric and the Powell conjugate gradient algorithm with a convergence criterion of 0.001 kcal/mol. Partial atomic charges were calculated by the Gasteiger–Huckel method [47]. Consequently, all the 24 substituted phenoxy acetic acids were aligned according to their common substructure. Molecular alignment was affected with the field fit alignment [59] method function of SYBYL. After consistently aligning the molecules within a lattice that extended 4 Å units beyond the aligned molecules in all directions with a grid space size of 2 Å, a probe sp3 carbon atom with a net charge of + 1 and van der Waals radius of 1.52 Å was employed. The five similarity indices in CoMSIA, i.e., steric, electrostatic, hydrophobic, H-bond donor, and H-bond acceptor descriptors were calculated and the fields generated were scaled by the CoMSIA-STD method in SYBYL-X 2.1.1. Here, steric indices are related to the third power of the atomic radii, the electrostatic descriptors are derived from the atomic partial charges, the hydrophobic fields are derived from the atom-based parameters, and the H-bond donor and acceptor indices are obtained by a rule-based method based on the experimental results. In optimizing the CoMSIA performance, the most important parameter is how to combine the five fields in the CoMSIA model. To choose the optimal result, we systematically altered the combination of fields and chose the value that gave the best non-cross-validation, the smallest errors, and the largest F (Fischer’s covariance ratio) value. Finally, the model generated by combining the steric, electrostatic, hydrophobic, and hydrogen bond acceptor and hydrogen bond donor fields was selected as the best CoMSIA model, and the contours were analysed using this model. To derive the 3D-QSAR models, the CoMSIA descriptors were used as independent variables with the respective activity (lnGU) value as a dependent variable. Partial least-squares (PLS) [59] regression analysis was performed with the standard protocol implemented in the SYBYL package. The predictive ability of the models was evaluated by leave-one-out (LOO) cross-validation. The developed model was further evaluated by predicting activities of the external test set compounds.

## Conclusion

The present work led to the development of novel glitazones in the lights of concepts of rational design using various computational techniques. Based on the pharmacophore modelling results, we decided docking protocols keeping PPARγ as target protein to virtually screen the library of glitazones and finally subjected for molecular dynamic simulation studies. The molecular dynamic simulation for best protein–ligand complex helped to comprehend the dynamic behaviour of glitazones in physiological environment.** CPD03, 07, 08, 18, 19, 21** and** 24** are the candidate glitazones to investigate further as they produce significant glucose uptake activity. The structure activity relationship was derived via CoMSIA model considering glucose uptake activity, thereby indirectly correlating reported glitazones as PPARγ agonists.
